# Gut microbiota modulation with long-chain corn bran arabinoxylan in adults with overweight and obesity is linked to an individualized temporal increase in fecal propionate

**DOI:** 10.1186/s40168-020-00887-w

**Published:** 2020-08-19

**Authors:** Nguyen K. Nguyen, Edward C. Deehan, Zhengxiao Zhang, Mingliang Jin, Nami Baskota, Maria Elisa Perez-Muñoz, Janis Cole, Yunus E. Tuncil, Benjamin Seethaler, Ting Wang, Martine Laville, Nathalie M. Delzenne, Stephan C. Bischoff, Bruce R. Hamaker, Inés Martínez, Dan Knights, Jeffrey A. Bakal, Carla M. Prado, Jens Walter

**Affiliations:** 1 Department of Agricultural, Food & Nutritional Science, University of Alberta, Edmonton, AB T6G 2E1 Canada; 2 Department of Medicine, University of Alberta, Edmonton, AB T6G 2E1 Canada; 3 School of Life Science, Northwestern Polytechnical University, Xi’an, 710072 People’s Republic of China; 4 College of Animal Sciences, Zhejiang University, Hangzhou, 310058 People’s Republic of China; 5 Food Engineering Department, Ordu University, 52200 Ordu, Turkey; 6 Whistler Center for Carbohydrate Research and Department of Food Science, Purdue University, West Lafayette, IN 47907 USA; 7 Institute of Nutritional Medicine, University of Hohenheim, 70593 Stuttgart, Germany; 8 Patient Health Outcomes Research and Clinical Effectiveness Unit, University of Alberta, Edmonton, AB T6G 2E1 Canada; 9 Centre de Recherche en Nutrition Humaine Rhône-Alpes and Centre Européen Nutrition Santé, 69310 Pierre Bénite, France; 10 Metabolism and Nutrition Research Group, Louvain Drug Research Institute, Université catholique de Louvain, 1200 Brussels, Belgium; 11 Department of Computer Science and Engineering, University of Minnesota, Minneapolis, MN 55455 USA; 12 BioTechnology Institute, University of Minnesota, Saint Paul, MN 55455 USA; 13 Department of Biological Sciences, University of Alberta, Edmonton, AB T6G 2E1 Canada; 14 School of Microbiology, Department of Medicine, and APC Microbiome Institute, University College Cork–National University of Ireland, Cork, T12 YT20 Ireland

**Keywords:** Arabinoxylan, Dietary fiber, Gut microbiota, Inter-individual variability, Overweight adults, Short-chain fatty acids

## Abstract

**Background:**

Variability in the health effects of dietary fiber might arise from inter-individual differences in the gut microbiota’s ability to ferment these substrates into beneficial metabolites. Our understanding of what drives this individuality is vastly incomplete and will require an ecological perspective as microbiomes function as complex inter-connected communities. Here, we performed a parallel two-arm, exploratory randomized controlled trial in 31 adults with overweight and class-I obesity to characterize the effects of long-chain, complex arabinoxylan (*n* = 15) at high supplementation doses (female: 25 g/day; male: 35 g/day) on gut microbiota composition and short-chain fatty acid production as compared to microcrystalline cellulose (*n* = 16, non-fermentable control), and integrated the findings using an ecological framework.

**Results:**

Arabinoxylan resulted in a global shift in fecal bacterial community composition, reduced α-diversity, and the promotion of specific taxa, including operational taxonomic units related to *Bifidobacterium longum*, *Blautia obeum*, and *Prevotella copri*. Arabinoxylan further increased fecal propionate concentrations (*p* = 0.012, Friedman’s test), an effect that showed two distinct groupings of temporal responses in participants. The two groups showed differences in compositional shifts of the microbiota (*p* ≤ 0.025, PERMANOVA), and multiple linear regression (MLR) analyses revealed that the propionate response was predictable through shifts and, to a lesser degree, baseline composition of the microbiota. Principal components (PCs) derived from community data were better predictors in MLR models as compared to single taxa, indicating that arabinoxylan fermentation is the result of multi-species interactions within microbiomes.

**Conclusion:**

This study showed that long-chain arabinoxylan modulates both microbiota composition and the output of health-relevant SCFAs, providing information for a more targeted application of this fiber. Variation in propionate production was linked to both compositional shifts and baseline composition, with PCs derived from shifts of the global microbial community showing the strongest associations. These findings constitute a proof-of-concept for the merit of an ecological framework that considers features of the wider gut microbial community for the prediction of metabolic outcomes of dietary fiber fermentation. This provides a basis to personalize the use of dietary fiber in nutritional application and to stratify human populations by relevant gut microbiota features to account for the inconsistent health effects in human intervention studies.

**Trial registration:**

Clinicaltrials.gov, NCT02322112, registered on July 3, 2015.

Video Abstract

## Background

Epidemiologic studies consistently associate dietary fiber consumption with a reduced incidence of obesity-associated pathologies [1, 2]. In large-scale observational studies, whole grains and cereal-derived fibers (e.g., arabinoxylan and β-glucan) showed stronger associations with reduced risk of developing cardiovascular disease, type II diabetes, gastrointestinal cancers, and of all-cause mortality when compared to other fiber sources [3, 4]. A substantial body of animal research further consolidated the mechanisms by which fiber reduces metabolic pathologies [5]. Despite these convincing associations, findings obtained from human dietary intervention trials aimed to improve metabolic risk markers by supplementing isolated dietary fibers remain inconsistent [6], possibly due to an individualized clinical response [7, 8].

Owing to their chemical structure, dietary fibers resist digestion in the small intestine and reach the colon where they become substrates for the gut microbiota. The microbial fermentation of fiber to short-chain fatty acids (SCFAs) has been implicated in the prevention of obesity-associated pathologies [9]. Propionate and butyrate are two SCFAs that are especially relevant, as they have been linked to beneficial immunological and metabolic effects [10]. Intervention studies with arabinoxylan isolated from wheat endosperm, for instance, have demonstrated increased fecal concentrations of both butyrate and propionate [11]. Dietary fibers can further modulate gut microbiota composition in a structure-dependent way through the enrichment of bacterial taxa that utilize the substrate and tolerate or benefit from the environmental changes caused by fiber fermentation [5, 12]. For example, dietary interventions with short-chain fractions of arabinoxylan resulted in an enriched abundance of bacterial species that can either utilize arabinoxylan oligosaccharides (AXOS) directly (e.g., *Bifidobacterium adolescentis* and *Bifidobacterium longum*) or benefit from metabolic by-products released during AXOS degradation (e.g., *Anaerobutyricum hallii* and *Faecalibacterium prausnitzii*) [13]. Although fiber-induced alterations to the gut microbiota are significant, the effects are also highly individualized [7], and this variability might have clinical ramifications that could explain the individualized clinical responses [14].

To understand the individualized response of the gut microbiota to dietary fiber, an ecological perspective is required, as fiber fermentation is determined by complex inter-species interactions between members of the gut microbiota [15]. The process is often based on a cross-feeding cascade, where primary degraders that access the fiber provide break-down products (oligosaccharides, disaccharides, and monosaccharides) to other microbes, and metabolites that result from the fermentation of these products also serve as substrates [16]. Inter-individual variation in gut microbiota composition may result from the absence of “keystone species” that initiate the degradation of recalcitrant fibers [17], differences in unrelated species with similar ecological functions that compete for the same substrate [18], or variation in strains of the same species that differ in their capacity to metabolize the substrate [19]. These compositional variations likely determine both the competitive and co-operative relationships between community members that form trophic networks, some of which organize into ecological “guilds” that collaborate to degrade complex fibers [20]. Although inter-individual variation in the response of the gut microbiota to fiber can influence metabolite outputs relevant to health (i.e., propionate or butyrate) [21], this topic, and the underlying ecological principles, have received little attention.

The objective of this study was to apply an ecological framework to characterize the compositional and metabolic responses of the human gut microbiota to a long-chain arabinoxylan isolated from corn bran compared to a fiber that is not fermented by the gut microbiota (microcrystalline cellulose, MCC). We further assessed whether nutritional and microbiota-related factors could explain the variable responses observed among individuals.

## Results

### Subject characteristics and protocol adherence

**Fig. 1:**
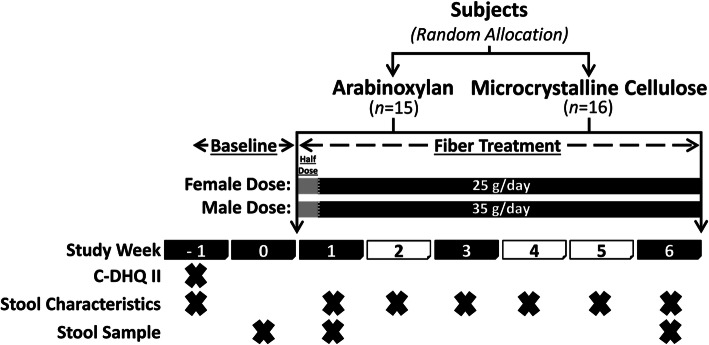
Study design. Shaded study week blocks indicate a scheduled clinic visit. The “X” indicates the task was completed during the study week. C-DHQ II, Canadian diet history questionnaire II; stool characteristics, self-reported stool consistency and bowel movement frequency

To compare the effects of arabinoxylan and MCC, we conducted a 6-week, parallel two-arm, exploratory, randomized controlled trial in individuals with overweight and class-I obesity, where females received 25 g/day and males 35 g/day of either fiber (Fig. 1). Of the 38 subjects enrolled and randomized to an intervention arm, seven withdrew from the study (in the arabinoxylan group, three experienced challenges consuming the supplement and one reported constipation; in the MCC group, two withdrew due to personal reasons and one due to constipation) and were, therefore, excluded from analyses (Additional file 1: Fig. S1). Subjects that completed the study protocol (*n* = 31) included 21 females and 10 males, aged 32.9 ± 8.5 years with a body mass index (BMI) of 28.7 ± 2.3 kg/m^2^. No differences in age, sex, or BMI were detected between the intervention groups at baseline (Additional file 2: Table S1). Overall, protocol adherence, assessed by the amount (weight) of returned supplement, was 94.7 ± 6.5% and 95.0 ± 5.6% in the arabinoxylan and MCC arms, respectively.

### Effect on the composition of the fecal microbiota

#### Fecal microbiota diversity

**Fig. 2:**
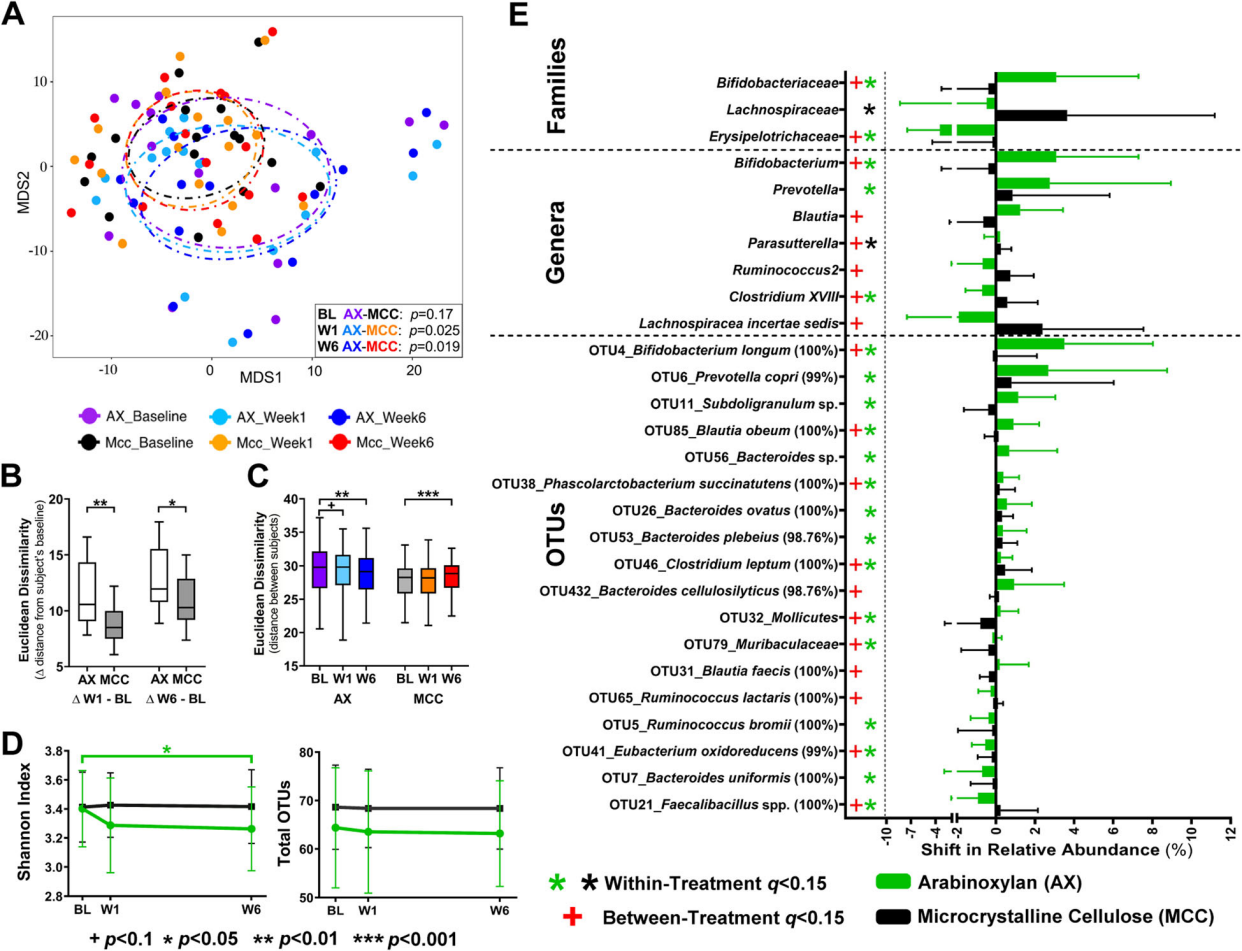
Arabinoxylan alters the global composition of fecal bacterial communities and induces distinct shifts in taxa. **a** Non-metric multidimensional scaling (NMDS) plot based on Euclidean distance metrics of arabinoxylan and microcrystalline cellulose groups at each time point (inter-subject β-diversity) showing changes in the distance between subjects over time. Euclidean distances **b** between fecal microbiotas of subjects at each study time point (inter-subject) and **c** between each subject’s fecal microbiota at baseline and during W1 and W6 of treatment (intra-subject). **d** α-Diversity (displayed as Shannon index and total OTUs) of the fecal microbiotas of subjects at each time point. **e** Absolute change (ΔW6–BL) in relative abundance of bacterial taxa affected by the dietary intervention. Data analyzed using PERMANOVA for **a**, GEE models (with Bonferroni correction) for **b** and **d**, and Mann–Whitney tests for **c**. For **e**, data were analyzed using either Wilcoxon tests to assess within-group changes relative to baseline, or Mann–Whitney tests to assess between-group changes (i.e., AX vs. MCC; with FDR correction). β-diversity and compositional data were reported as mean ± SD, and centered log-ratio transformed prior to the statistical analyses. *BL* baseline, *OTU* operational taxonomic unit, *W1* week 1, *W6* week 6

Non-metric multidimensional scaling analysis of Euclidean distances between subjects based on centered log-ratio (CLR)-transformed operational taxonomic unit (OTU) data showed that the two treatment groups harbored bacterial communities that could not be differentiated at baseline (*p* = 0.17, permutational multivariate analysis of variance [PERMANOVA]; Fig. 2a). One-week supplementation with arabinoxylan altered the global fecal bacterial community, which became significantly different from the fecal microbiota of subjects receiving MCC (*p* = 0.025). This effect was maintained until the end of the fiber intervention (*p* = 0.019). These changes occurred by arabinoxylan inducing temporal shifts in fecal microbiota composition, determined as the average β-diversity between the individual’s treatment and baseline samples, which were significantly larger when compared to the MCC group (*p* ≤ 0.015 Mann–Whitney test; Fig. 2b). In addition, while MCC increased inter-individual differences (β-diversity between subjects; *p* < 0.001, generalized estimated equation [GEE] model), arabinoxylan reduced it (*p* = 0.003, Fig. 2c).

Analysis of α-diversity showed that arabinoxylan reduced fecal bacterial diversity (Shannon’s index) (*p* = 0.036, GEE model; Fig. 2d) but not richness (total OTUs) after 6 weeks of supplementation. Overall, these findings showed that while the non-fermentable MCC had no detectable effects on measures of bacterial diversity, arabinoxylan altered the global bacterial community within 1 week, inducing temporal shifts in composition and a reduction of both inter-individual variation and α-diversity.

#### Effect on the relative abundance of bacterial taxa and co-abundance response groups

Neither arabinoxylan nor MCC altered microbiota composition at the phylum level. At lower taxonomic levels, changes in the relative abundance of two bacterial families were detected at 6 weeks of arabinoxylan relative to baseline and MCC, namely an increase in *Bifidobacteriaceae* (*q* = 0.04, Wilcoxon test; Fig. 2e, Additional file 3: Table S2) and a decrease in *Erysipelotrichaceae* (*q =* 0.004). At the genus level, arabinoxylan increased the genera *Bifidobacterium* and *Prevotella* when compared to both baseline and MCC, and enriched *Blautia* when compared to MCC. OTU level analysis revealed that 15 OTUs changed during arabinoxylan treatment relative to baseline (henceforth referred to as “significant OTUs”). In particular, OTUs related to *Bifidobacterium longum* (OTU4), *Prevotella copri* (OTU6), *Bacteroides plebeius* (OTU53), *Bacteroides* sp. (OTU56), *Bacteroides ovatus* (OTU26), *Phascolarctobacterium succinatutens* (OTU38), *Blautia obeum* (OTU85), *Subdoligranulum* sp. (OTU11), *Clostridium leptum* (OTU46), *Mollicutes* (OTU32), and *Muribaculaceae* (OTU79) (*q* < 0.15) became enriched, while OTUs related to *Ruminococcus bromii* (OTU5), *Eubacterium oxidoreducens* (OTU41), *Bacteroides uniformis* (OTU7), and *Faecalibacillus* spp. (OTU21) declined in relative abundance. Supplementation with MCC only increased the family *Lachnospiraceae* and the genus *Parasutterella* (*q* = 0.117). Numerically, the dominant compositional effects of arabinoxylan were, to a large degree, specific to *B*. *longum* (OTU4) and *P*. *copri* (OTU6), as these taxa increased in relative abundance by an average of 3.5% (46-fold) and 2.7% (4-fold), while other OTUs increased by ≤ 1.1%.

**Fig. 3:**
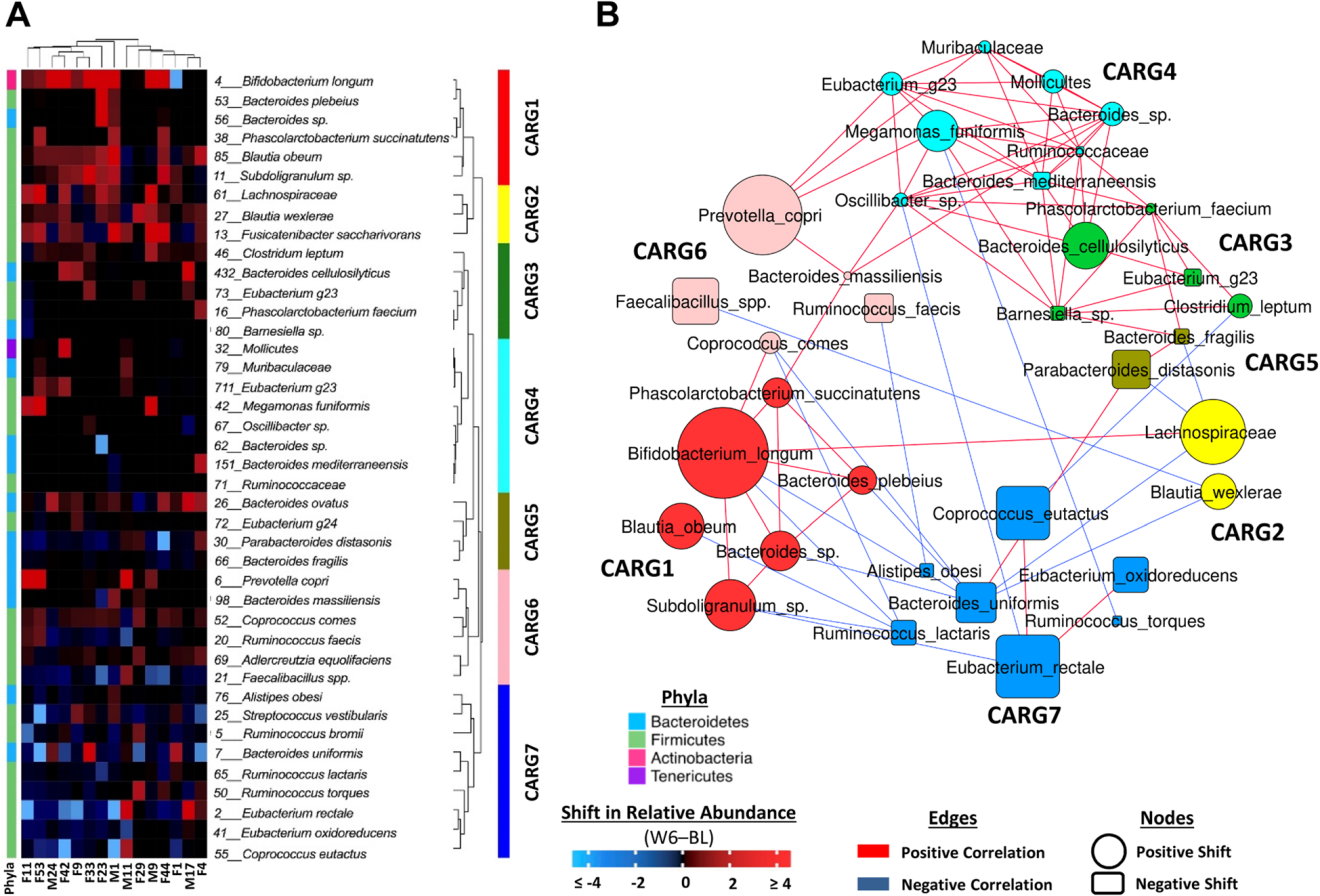
Identification of co-abundance response groups (CARGs) during arabinoxylan supplementation. **a** Heatmap shows the change (ΔW6–BL) in relative abundance of 41 OTUs affected by arabinoxylan (*p* < 0.1, Wilcoxon test). The hierarchical dendrogram shows clustering of centered log-ratio (CLR) transformed OTUs (rows) based on Spearman’s correlation distances by the complete-linkage clustering algorithm, and then grouped on the dendrogram into seven CARGs by PERMANOVA (*p* < 0.05). Subjects (columns) clustered based on Euclidean distances. Colors from blue to red indicate the direction and magnitude of change. **b** Co-response network analysis. Each node represents an OTU, where the size is proportional to the change (ΔW6–BL) in relative abundance, the shape indicates direction of change (positive: circle; negative: square), and the color references the respective CARG to which it was clustered. Lines between nodes represent significant positive (red line) or negative (blue line) Spearman’s correlations (*r*_s_ values ≥ 0.5 or ≤ − 0.5 and *q* values < 0.05). *BL* baseline, *OTU* operational taxonomic unit, *W6* week 6

In an attempt to identify groups of co-operating species that could function as ecological guilds in the degradation of arabinoxylan, we adapted a clustering approach conceptually similar to that described by Tong et al. [22]. Instead of absolute proportions of taxa, we used arabinoxylan-induced shifts to identify clusters of species whose responses were inter-correlated. This analysis revealed a total of seven co-abundance response groups (CARGs) (Fig. 3a), five of which showed statistically significant responses to arabinoxylan, while none responded to MCC (Additional file 3: Table S2). The CARG that showed the largest increase in relative abundance was CARG1 (*p* = 0.0003, Wilcoxon test), which consisted of six out of the eleven OTUs that increased through arabinoxylan (Fig. 3b). Among those six OTUs, *B*. *longum* (OTU4) exhibited the largest shift and showed significant connections to all but one member of CARG1 (*r*_s_ > 0.5, *q* < 0.05; Spearman’s correlations using permutation tests), suggesting arabinoxylan may be degraded through co-operative interactions between these taxa. In CARG6, *P*. *copri* (OTU6) exhibited the largest response, but only showed one strong connection with another member of the CARG, *Bacteroides massiliensis* (OTU98; *r*_s_ = 0.71, *q* = 0.007), which suggests that *P*. *copri* might act to a larger degree independently to degrade arabinoxylan (Fig. 3b). The majority of taxa that decreased during arabinoxylan consumption, particularly *B*. *uniformis* (OTU7), clustered within CARG7 and showed negative correlations with taxa of CARG1, CARG2, and CARG6, suggesting competitive or antagonistic interactions.

#### Temporal responses of OTUs and CARGs

**Fig. 4:**
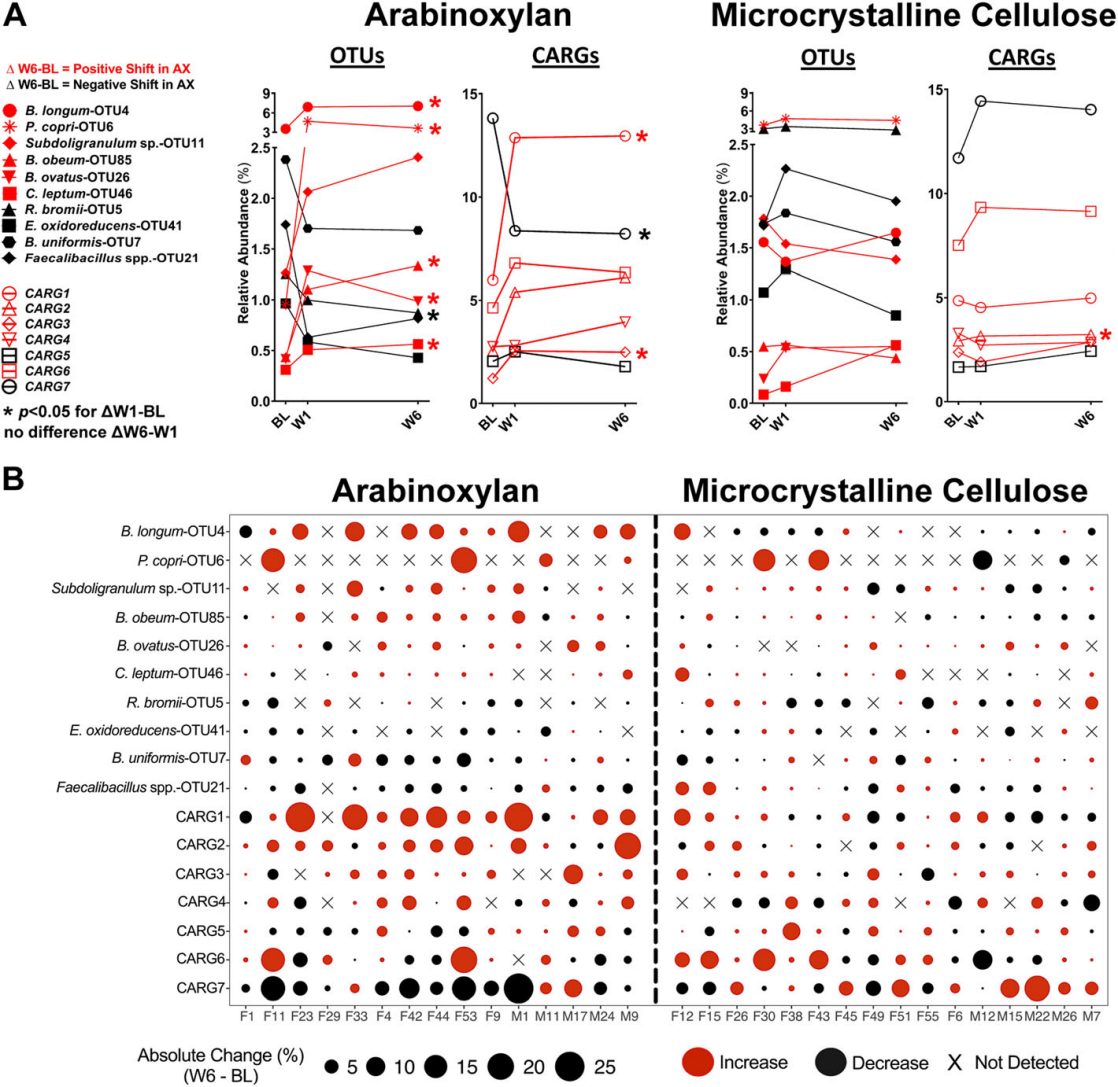
Temporal and individualized responses of the OTUs and CARGs affected by arabinoxylan and microcrystalline cellulose. **a** Plots show the temporal response of the ten most abundant OTUs (detected in > 25% of subjects) and the seven CARGs. Centered log-ratio transformed data were analyzed by Friedman’s test (with Dunn’s correction) to assess within-group changes between time points (i.e., ΔW1–BL and ΔW6–W1). **b** Bubble plot shows individualized differences (ΔW6–BL) in relative proportions of the ten most abundant OTUs (percentage of total microbiota composition) and CARGs (sum of OTUs) detected after 6 weeks of arabinoxylan and microcrystalline cellulose supplementation. The size of the bubble is proportional to the change in abundance relative to baseline, while the color of the bubble represents the direction of the change (red: increase; black: decrease). The “X” indicates that the OTU was either undetected or the change was < 0.02% relative abundance. *BL* baseline, *CARG* co-abundance response group, *OTU* operational taxonomic unit, *W1* week 1, *W6* week 6

To determine if short- and long-term treatment with arabinoxylan and MCC differed in their effects on the fecal microbiota, we compared shifts from baseline to week 1 (W1) with those from baseline to week 6 (W6); however, there were no detectable differences between the two time frames (*q* > 0.25, Wilcoxon test, data not shown). In addition, comparison of baseline, W1, and W6 values by Friedman’s test indicated that the effects of arabinoxylan occur rapidly (within 1 week), with no further detectable changes at 6 weeks (Fig. 4a). Considering these findings, analyses on compositional changes were performed with W6 data unless otherwise stated.

#### Inter-individual variation in responses to arabinoxylan

Bacterial shifts in response to arabinoxylan and their magnitude were highly individualized (Fig. 4b). For instance, absolute increases in relative abundance ranging from 5 to 13% (2- to 429-fold change) were detected in seven subjects for the OTU classified as *B*. *longum* (OTU4), while other subjects showed either a much smaller increase, a decrease, or the species was undetectable. OTUs related to *B*. *obeum* (OTU85), *Subdoligranulum* sp*.* (OTU11), *B*. *ovatus* (OTU26), and *C*. *leptum* (OTU46) were enriched by arabinoxylan in around two-thirds of the subjects. Less frequently enriched were OTUs classified as *P*. *copri* (OTU6), *B*. *plebeius* (OTU53), and *Bacteroides* sp. (OTU56). *P*. *copri* (OTU6) responded in only four subjects, but effects were large, with the species expanding beyond 10% (2- to 7-fold change) of the total bacterial community in three subjects.

To determine drivers of these individualized responses, we used multiple linear regression (MLR) analyses to test if responses in OTUs that showed, numerically, the largest shifts (*P*. *copri*, *B*. *longum*, *B*. *obeum*, and *Subdoligranulum* sp.) and in CARGs with significant responses (CARGs 1, 2, 3, 6, and 7) could be predicted by baseline diet or microbiota composition. Baseline microbiota (all OTUs and significant OTUs) and diet variables were first reduced in their dimensionality by principal component analysis (PCA) and then treated as predictors. This analysis revealed that individualized responses of bacterial taxa and CARGs to arabinoxylan and MCC could not be predicted by baseline diet or microbiota composition (*q* > 0.05; Additional file 4: Fig. S2).

### Effect on stool characteristics and bowel movements

While fecal moisture content was not changed by either fiber (*q* > 0.2, Wilcoxon test; Additional file 5: Table S3), subjects consuming arabinoxylan reported softer stool consistencies when compared to subjects consuming MCC (treatment effect *p* = 0.049, GEE model; Additional file 6: Fig. S3a). Both arabinoxylan and MCC led to an increase in bowel movements relative to baseline (*p* < 0.05, GEE model; Additional file 6: Fig. S3b), with no difference detected between treatment groups (treatment effect *p* = 0.8).

### Effect on fecal pH and SCFAs

Fecal pH and SCFA concentrations did not change after 6 weeks of either fiber treatment (*q* > 0.2, Wilcoxon test; Additional file 5: Table S3). Considering that absolute concentrations of fecal SCFAs are affected by their absorption in the gut [23], we additionally assessed changes in the percentages of acetate, propionate, and butyrate relative to total SCFA concentrations at W6, which has been previously shown to vary little across colonic regions [24]. This analysis revealed an increase in the percentage of propionate produced through arabinoxylan when compared to MCC (*q* = 0.07, Mann–Whitney test) and a reduction in the percentage of butyrate relative to baseline (*q* = 0.13, Wilcoxon test), although differences in butyrate were not detected when compared to MCC (*q* = 0.31). Further investigation of the ratio between propionate and butyrate showed an increase in propionate relative to butyrate when compared to baseline (*q* = 0.06) and MCC (*q* = 0.07), suggesting that arabinoxylan supplementation directed the output of SCFAs in favor of propionate.

**Fig. 5:**
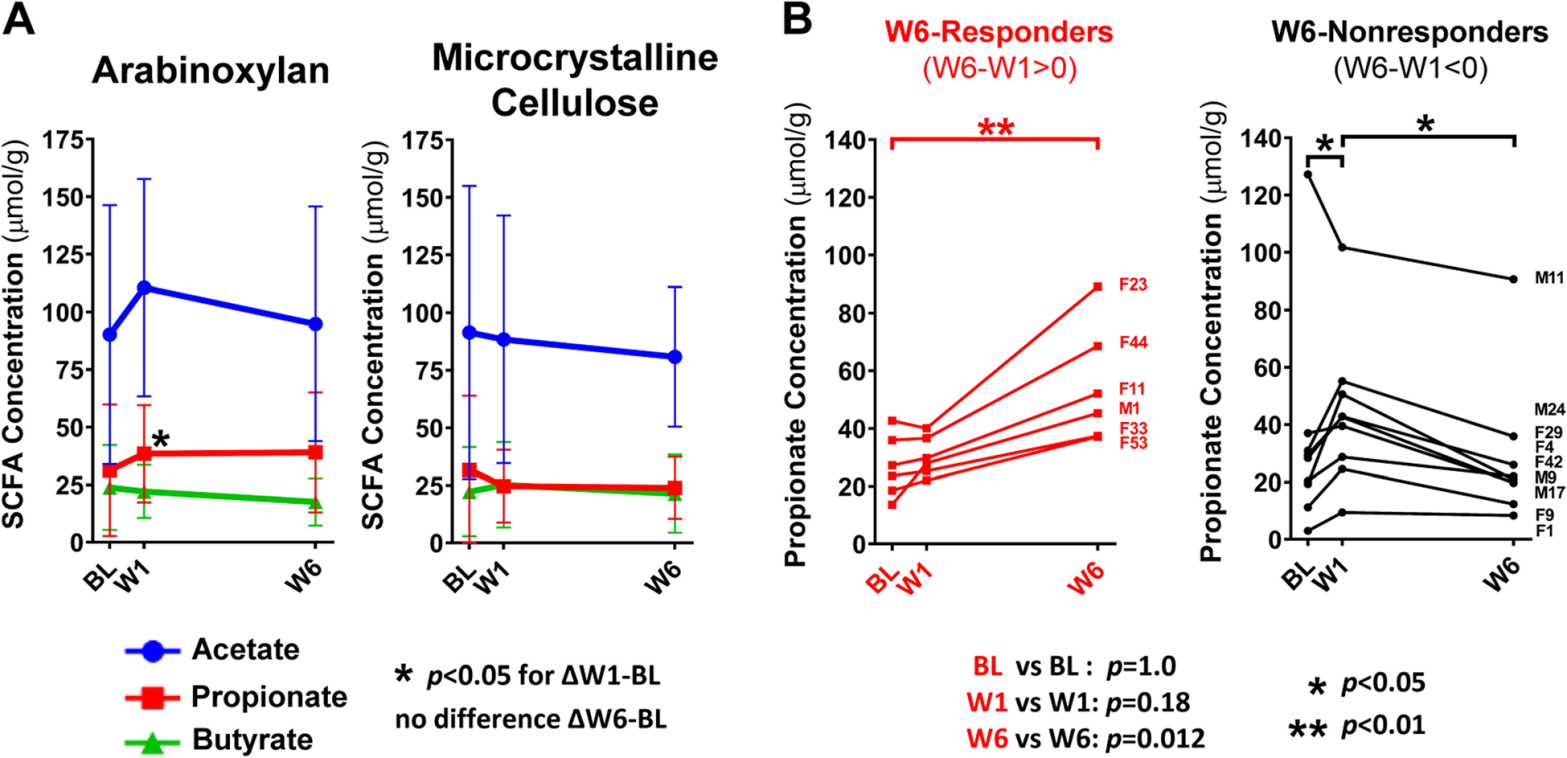
Temporal and individualized output of fecal SCFAs in response to arabinoxylan and microcrystalline cellulose supplementation. **a** Line plots show the temporal response of acetate, propionate, and butyrate; reported as mean ± SD. **b** Individualized temporal propionate response of W6-responders (red) and W6-nonresponder (black) (grouped based on ΔW6–W1). Data analyzed for **a** and **b** using Friedman test (with Dunn’s correction) to assess within-group changes between time points, and for **b** using Mann–Whitney tests to assess differences between-group at each time point. *BL* baseline, *CARG* co-abundance response group, *OTU* operational taxonomic unit, *SCFA* short-chain fatty acid, *W1* week 1, *W6* week 6

Characterization of the temporal response in the three primary SCFAs also showed an increase in fecal propionate concentrations by arabinoxylan at W1 (*p* = 0.01, Friedman’s test) (Fig. 5a). Although propionate concentrations remained elevated at W6, this increase was not statistically significant when compared to baseline (*p* = 0.15). This loss of significance was caused by an increase in the inter-individual variation at W6 (Fig. 5b). Visual evaluation of the individualized temporal response of propionate to arabinoxylan revealed clear separation of subjects into two distinct patterns (Fig. 5b). Based on the direction of change from W1 to W6 (i.e*.,* positive or negative), subjects were grouped into “W6-responders” (Δ W6–W1 > 0) and “W6-nonresponders” (Δ W6–W1 < 0). In general, W6-responders showed a higher output of propionate at W6 (*p* = 0.0045, Friedman’s test) but not at W1, while the opposite is seen in W6-nonresponders (*p* = 0.014). The two groups differed by propionate concentrations at W6 (*p* = 0.012, Mann–Whitney test).

### W6-propionate responders and nonresponders differ in their microbiota response to arabinoxylan

**Fig. 6:**
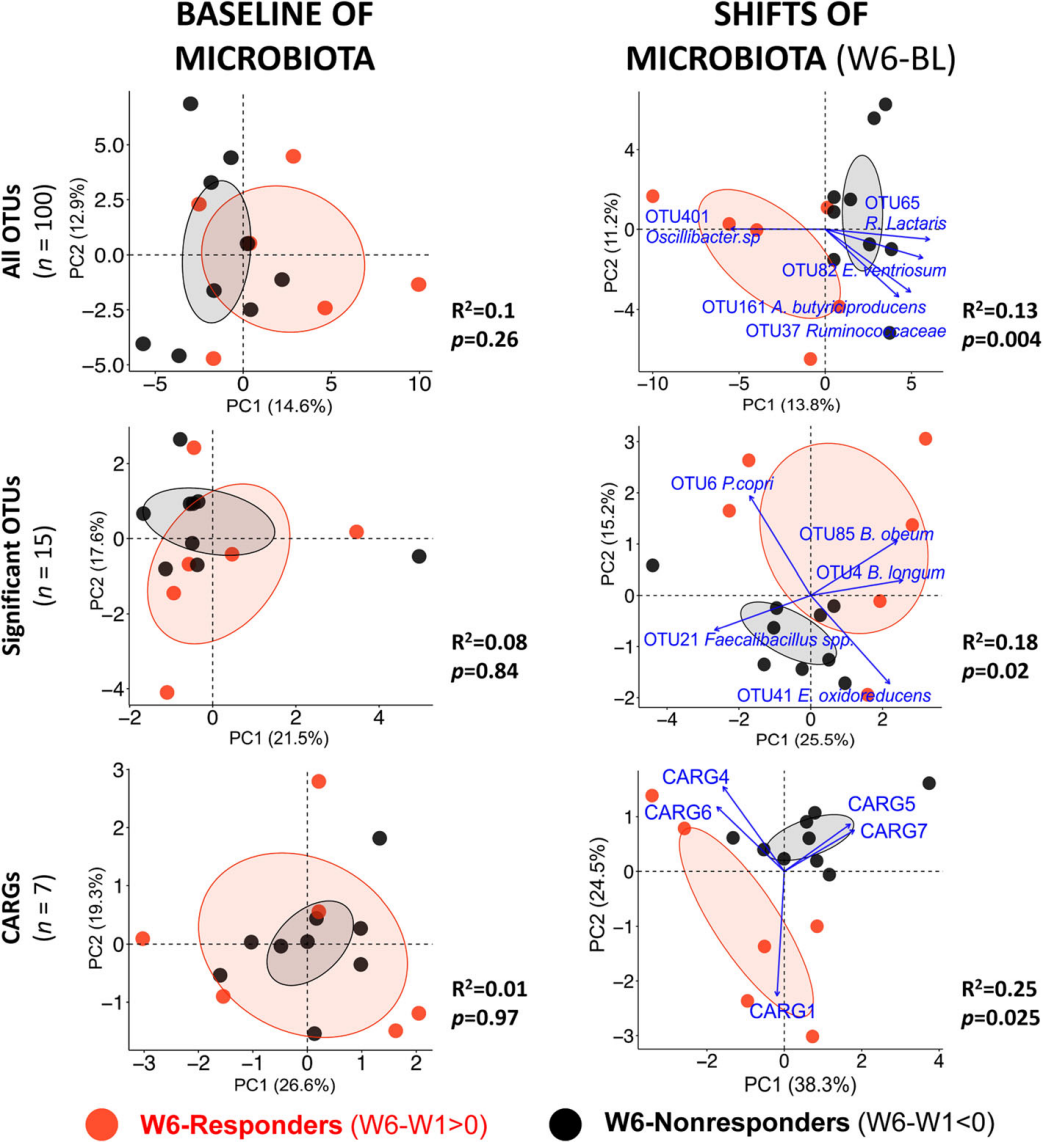
The individualized temporal propionate response to arabinoxylan associates with compositional responses in the fecal microbiota. Principal component analysis plots based on Euclidean distance comparing the relative abundance of fecal microbiota, both at baseline and arabinoxylan-induced shifts (ΔW6–baseline), between W6-responders (red) and W6-nonresponders (black). Microbiota variables (i.e., OTU or CARG) that contributed the most to inter-subject variation were shown as vectors on the plot when statistical significances were determined by PERMANOVA (*p* < 0.05). *CARG* co-abundance response group, *OTU* operational taxonomic unit, *W1* week 1, *W6* week 6

Microbiota compositional (baseline and shifts) and diet data were ordinated using PCA, and then differences between W6-propionate responders and nonresponders were tested using PERMANOVA. This analysis revealed that the bacterial communities of W6-responders were indistinguishable from W6-nonresponders at baseline but differed in their response to arabinoxylan (ΔW6-baseline; Fig. 6). This was detected if analysis was based on all OTUs (*p* = 0.004), the 15 significant diet-responsive OTUs (*p* = 0.025), or the seven CARGs (*p* = 0.025). In contrast, neither baseline microbiota composition (Fig. 6) nor dietary factors (Additional file 7: Fig. S4a) separated according to W6 response (*p* > 0.1). In addition, comparing W6-responders and W6-nonresponders in terms of their baseline total grain, whole grain, and total fiber consumption or their stool consistency and bowel movement frequency during treatment did not reveal any differences either (*p* > 0.1, Mann–Whitney test) (Additional files 6 and 7: Fig. S3c and Fig. S4b). Together, these findings indicate that the temporal response in fecal propionate concentrations is primarily associated with the shifts in the microbiota and not baseline microbiota composition or diet.

### Individualized SCFA responses can be predicted by gut microbiota features

**Fig. 7:**
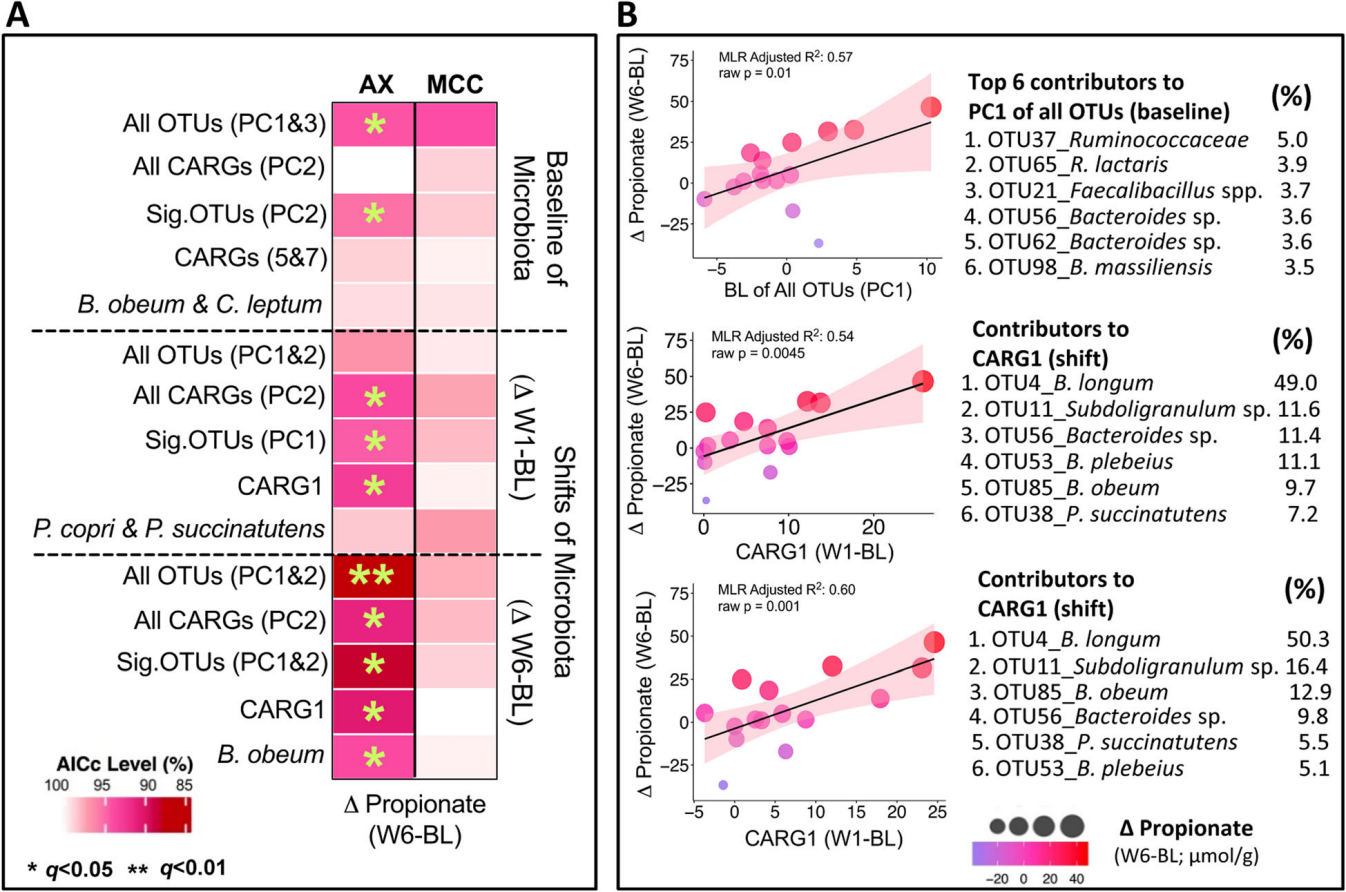
Individualized arabinoxylan-induced propionate responses could be explained by baseline gut microbiota composition and microbiota shifts. **a** Heatmap shows the linear associations between the individualized propionate response (ΔW6–BL; dependent variable; columns) and microbiota profiles (BL, ΔW1–BL, ΔW6–BL; predictors; rows). Cells represent individual multiple linear regression models (with FDR correction) that assess whether the predictors explain the individualized propionate response. Multivariate microbiota data were simplified into principal component (PC) variables PC1, PC2, and PC3 prior to analysis. Each model contained the best one or two predictors of PCs, individual CARGs, or significant OTUs selected by stepwise regression. All models were adjusted by fiber dose/sex. Colors from white to red indicate relative AICc (corrected Akaike information criterion) values calculated by $\frac{\mathrm{AICc}\ \mathrm{value}}{\mathrm{Highest}\ \mathrm{AICc}\ \mathrm{value}}\mathrm{x}\ 100$. Lower AICc values (red) indicate higher quality models. **b** Scatter plots show the linear relationship between propionate responses (ΔW6–BL) and either the baseline contribution of all OTUs to PC1 or the shifts of CARG1. Color and size of each point indicate propionate response magnitude and the shaded area specifies the 95% confident interval. The top six OTUs that contributed the most to either PC1 of all OTUs or CARG1 are further provided. *AX* arabinoxylan, *BL* baseline, *CARG* co-abundance response group, *MCC* microcrystalline cellulose, *OTU* operational taxonomic unit, *W1* week 1, *W6* week 6

As with compositional responses, gut microbiota functional responses to fiber interventions have been shown to be individualized [7, 25, 26], but what drives this variation is poorly understood. We applied MLR to determine whether fecal SCFA responses could be explained by stool consistency and bowel movement frequency, diet, or microbiota-related factors, and then compared the quality of the models using corrected Akaike information criterion (AICc) values (where lower values mean higher quality). These analyses revealed that the W6 SCFA response to arabinoxylan could be predicted by the fecal microbiota (Fig. 7; Additional file 8: Fig. S5) but not by baseline diet, stool consistency, or bowel movement frequency reported during treatment (Additional file 9: Fig. S6a and Fig. S6b). The best models were achieved for propionate, especially when principal components (PCs) generated from W6 shifts of all OTUs were used as predictors (Fig. 7a; Additional file 10: Table S4). Models were of lower quality when W6 shifts of significant OTUs, CARGs, PCs of CARGs, or single OTUs were used, suggesting that global community measures exhibited stronger linear relationships with the propionate response than single or groups of taxa. Although the models that used baseline and W1 shifts of OTUs as predictors were of lower quality than those based on W6 shifts, they are still valid, showing *q* values less than 0.05 after Benjamini-Hochberg’s false discovery rate (FDR) correction. Linear relationships between propionate responses and significant predictors using baseline (PC1 of all OTUs) and shifts (CARG1) were further visualized using scatter plots (Fig. 7b), reaffirming the quality of the analysis, as a majority of subjects fall within the 95% confidence regions.

Significant models could also be designed for acetate and butyrate responses to arabinoxylan (Additional file 8: Fig. S5). Interestingly, in contrast to propionate, the best models to predict butyrate responses were achieved using shifts of a single OTU, *E*. *oxidoreducens* (OTU41), a known butyrate producer [27]. However, overall, the models for acetate and butyrate were of much lower quality than those for propionate. In summary, while individualized responses in SCFAs showed no association with diet, they could be predicted by microbiota shifts and baseline composition. In contrast to the analysis of the effects of arabinoxylan, not one single MLR model was found to be significant for MCC, indicating that the statistical approach based on MLR models did not detect any associations independent of fiber fermentation.

### Determining the role of bacterial taxa in propionate response

**Fig. 8:**
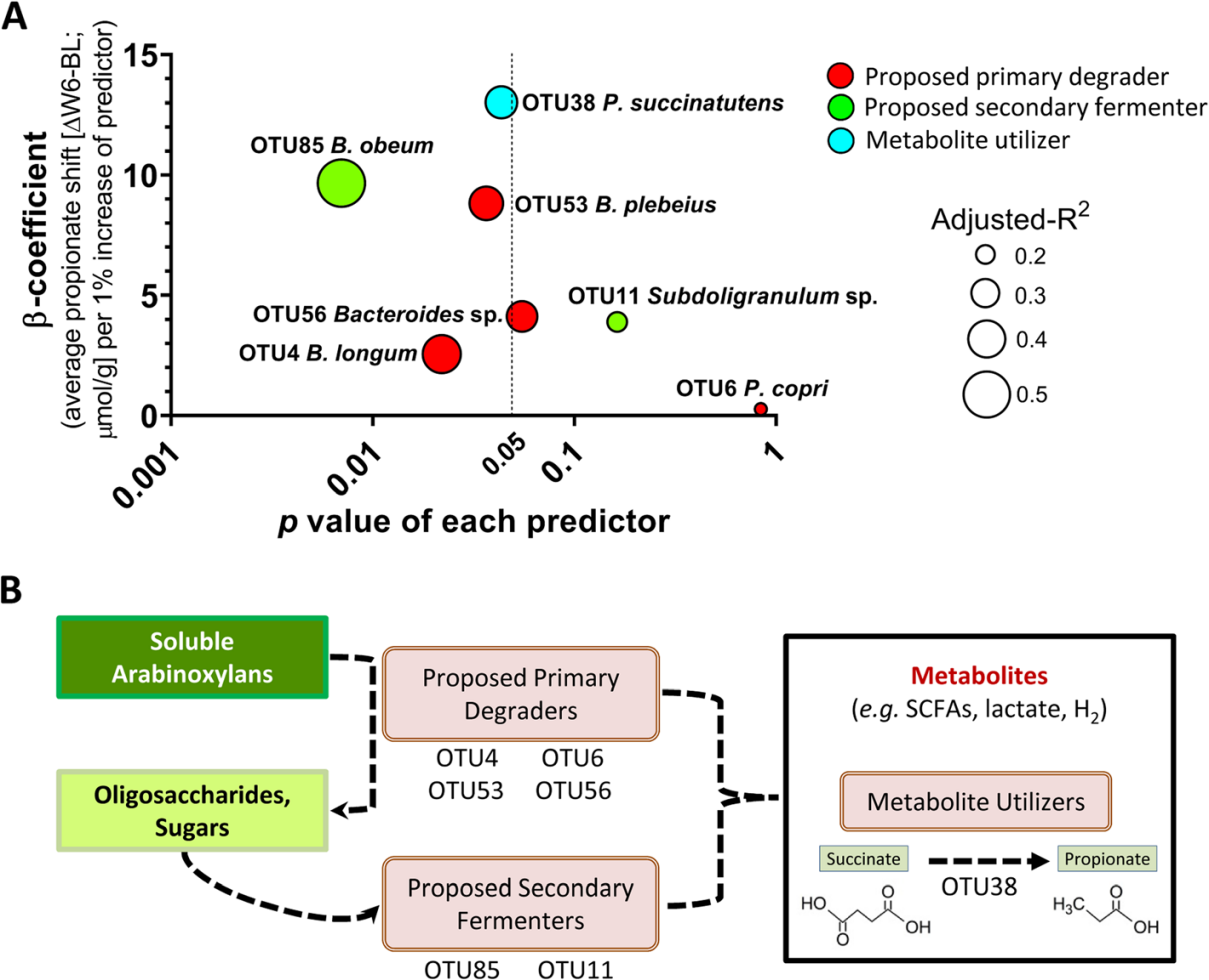
Relationship between propionate responses to arabinoxylan and proposed primary degraders, secondary fermenters, and metabolite utilizers. **a** Individual multiple linear regression models determine OTU responses (ΔW6–BL) that predict the fecal propionate response (ΔW6–BL). *Y*-axis shows the β-coefficient for each predictor, as in the average propionate response when OTU relative abundance increases 1%. *X*-axis shows the *p* value for each predictor. All models were adjusted by fiber dose/sex, where bubble size represents the adjusted-*R*^2^. **b** Proposed model of bacterial cross-feeding in the gut during degradation of complex, soluble arabinoxylans. *OTU* operational taxonomic unit

MLR analyses were applied to determine connections between arabinoxylan responding OTUs within CARGs 1 and 6, and fecal propionate concentrations (Fig. 8a). This analysis revealed that shifts in *P*. *copri* (OTU6) did not predict propionate responses, while *B*. *longum* (OTU4) and correlated taxa in CARG1 showed stronger linear relationships. The highest quality models were obtained with *B*. *obeum* (OTU85), *B*. *plebeius* (OTU53), and *P*. *succinatutens* (OTU38), all of which encode metabolic pathways for propionate production [28]. Such analysis provides a potential explanation for the metabolic interactions between proposed primary degraders, secondary fermenters, and metabolite utilizers that result in the promotion of propionate in response to arabinoxylan (Fig. 8b).

## Discussion

In the present study, we characterized the impact of a 6-week, high-dose corn bran arabinoxylan supplementation on the composition and function of the fecal bacterial community in healthy adults with overweight and class-I obesity. Arabinoxylan treatment changed community structure and induced specific shifts in the composition of the gut microbiota that manifested themselves after 1 week of treatment without further changes at W6. Arabinoxylan induced increases in propionate output. Both compositional and functional responses were highly individualized, with propionate responses showing two distinct temporal patterns. Compositional responses to arabinoxylan could not be predicted and functional responses were independent of stool consistency, bowel movement frequency, and baseline diet; however, baseline microbiota composition and especially the compositional shifts correlated with propionate responses. The non-fermentable MCC showed virtually no effect on gut microbiota composition or function.

An understanding of compositional and functional responses of the gut microbiota to changes in diet requires an ecological framework [15]. Arabinoxylan supplementation provides resources that can be used by microbes that possess the traits to either access the chemical structures directly or utilize public goods released during arabinoxylan degradation [15]. In our study, the dominant effects of arabinoxylan were directed toward two bacterial species, *B*. *longum* and *P*. *copri*, while nine additional OTUs showed smaller increases, including three *Bacteroides* species (e.g., *B*. *ovatus*, *B*. *plebeius*, and *Bacteroides* sp.). This high degree of specificity toward *B*. *longum* over other *Bifidobacterium* species is in agreement with other studies testing long-chain arabinoxylans [29–31] and genomic analyses that showed that genes encoding arabinoxylan-degrading glycosidase (e.g., β-xylosidase and α-arabinofuranosidase) are conserved only among *B*. *longum* strains [32, 33]. In contrast to the species-specific enrichment of *B*. *longum*, arabinoxylan enriched several species within the phylum Bacteroidetes that possess the genetic and functional traits necessary for accessing arabinoxylan [34–38]. Although arabinoxylan utilization is not universally conserved among the genera *Bacteroides* and *Prevotella*, the species *P*. *copri*, *B*. *ovatus*, *B*. *cellulosilyticus*, and *B*. *plebeius* have been shown to be xylanolytic [34–36, 38, 39] and possess polysaccharide utilization loci that encode for the xylan utilization system [34, 37, 40], thus providing an explanation for their enrichments in our study.

Interestingly, there were several consistent effects observed between the corn bran arabinoxylan used in our study and wheat bran extracted AXOS, such as increases in *Bifidobacterium longum*, *Prevotella copri*, *Bacteroides ovatus*, and *Blautia obeum* [13, 41]. However, in contrast to corn-bran arabinoxylan, AXOS seem to have lower specificity and promote multiple species of *Bifidobacterium* and *Prevotella*, as well as several additional genera (e.g., *Eubacterium* and *Roseburia*). This difference in specificity is likely attributed to variations in their structural features. Specifically, corn bran arabinoxylan exhibited a relatively high arabinose-to-xylose ratio of 0.56 and contained high amounts of galactose (9.7%), which suggests a heavily branched structure with complex side chains [42–44]. To access and utilize such complex structures, bacteria require a more extensive repertoire of proteins and hydrolases relative to what is needed for AXOS utilization, which are generally simpler in structure [45]. For instance, *B*. *adolescentis* has been shown to utilize simple AXOS both in monoculture [46] and during co-culture with *B*. *ovatus*, but not during co-culture on corn bran arabinoxylan [47].

Exploring the response of the bacterial community in the context of ecological guilds provides a more complete view on the interactions among the bacterial species in the degradation of arabinoxylan. This analysis showed the strongest response in CARG1 and CARG2. The response within CARG1 is dominated by *B*. *longum*, which showed strong connections to four out of five members within CARG1 (*B*. *plebeius*, *Bacteroides* sp., *P*. *succinatutens*, and *Subdoligranulum* sp.) and one member in CARG2 (an unclassified *Lachnospiraceae*), suggesting syntrophic relationships. *B*. *longum* has been shown to be a primary degrader of arabinoxylans [32, 33] that is able to cleave the complex arabinoxylan structure by soluble arabinoxylan-degrading glycosidase [48, 49]. This degradation could release xylan and AXOS (or even xylose, arabinose, and galactose) to xylan-utilizing *Bacteroides* species like *B*. *plebeius* [36, 37, 40] and putative secondary fermenters like *B*. *obeum* and *Subdoligranulum* sp. [29, 50, 51] (Fig. 8b). This cross-feeding would explain the strong positive associations between *B*. *longum* and the other OTUs within CARG1. In contrast, *P*. *copri* also increased and is likely a primary degrader of arabinoxylan [34, 39], but showed only one strong correlation within CARG6, suggesting the bacterium behaves “selfishly.” Our findings suggest that no singular “keystone species” initiates the degradation of arabinoxylan, as it has been described for type-III resistant starches [17]. Most likely, several primary degraders, including *B*. *longum*, *P*. *copri*, and certain *Bacteroides* species, assume this task.

The ecological connections described above provide a basis to understand the effects of arabinoxylan on microbiota metabolism and the increase in propionate. The specificity of long-chain arabinoxylans for propionate has been previously described [52, 53], and is affiliated with a higher presence of arabinose side-chains [3, 54]. Although *P*. *copri* is a primary degrader of arabinoxylan, the species did not predict propionate response in our study, which is in accordance with previous suggestions that the bacterium acts selfishly [55] and does not produce propionate [28]. Metabolic interactions appear more relevant within CARG1. Although *B*. *longum* is numerically the dominant responder within this CARG, it does not produce propionate itself and is a poor predictor of propionate responses (Fig. 8a). However, the enrichment of *B*. *longum* is strongly linked to species that possess metabolic pathways for propionate production (i.e., *B*. *obeum*, *P*. *succinatutens*, *B*. *plebeius*, and *Bacteroides* sp.) [56, 57], which are better predictors of propionate response. Although significant models were obtained with MLR using the single taxa of CARG1, the entire CARG1 was a better predictor of propionate shifts, indicating that groups of bacteria collaborate to produce propionate. Overall, our analyses on ecological guilds suggest co-operative and syntrophic interactions among *B*. *longum*, *B*. *obeum*, *P*. *succinatutens*, and some *Bacteroides* species in the degradation of arabinoxylan to produce propionate, while *P*. *copri* displays a more competitive phenotype during arabinoxylan degradation.

Although our study revealed significant effects of arabinoxylan on microbiota composition and propionate production, these effects displayed a high degree of individuality. In terms of taxa, this might be driven by the inter-individual differences in baseline microbiota composition and diet [7]. Although the responses of *P*. *copri* were strictly linked to the presence of the species at baseline, our MLR models showed no significant associations between baseline CLR-transformed abundances and individualized responses. However, some models showed *p* values below 0.001 before FDR correction, suggesting that associations between the compositional response to arabinoxylan and the baseline microbiota exist but could not be detected with the small sample size of our study. MLR analyses further showed that baseline reported dietary history could not predict arabinoxylan-induced shifts in bacterial taxa or CARGs. This might be reflective of the fact that diet is only one of many contributors to the variation of microbiomes [58, 59], although we cannot exclude that our small sample size and limitations in self-reported food frequency questionnaire data contributed to the lack of signal [60]. Therefore, future studies on the individualized response to fiber should be conducted with larger sample sizes, repeated dietary recalls or records, and whole metagenome sequencing to achieve higher resolution, strain-level distinctions that likely drive individuality.

Individuality was especially pronounced when looking at metabolite output. Our MLR analyses revealed that shifts in propionate output (∆W6-baseline) correlated with W6 shifts of the microbiota, and to a lesser degree W1 shifts and baseline composition, but not diet, stool consistency, or bowel movement frequency. Shifts in CARGs provided a better prediction than individual taxa indicate the importance of ecological guilds in fiber fermentation. However, models using the first two PCs generated from all OTUs, capturing 25% of the variance in the bacterial response, were of better quality than those using CARGs, demonstrating that propionate production is the result of a more complex trophic network that spans the wider bacterial community. This can potentially be explained by the well-recognized functional redundancy among distantly related members of the gut microbiota [28, 61], and the stochastic nature by which they assemble into communities [62]. Although it is often assumed that functional redundancy results in gut microbiomes that are more similar between individuals on a functional level, our findings on the propionate response to arabinoxylan clearly show that differences among individuals exist in terms of how they ferment a dietary fiber. The hierarchy by which factors predict propionate response found in our MLR analysis (single taxa < CARGs < PCs) supports an ecological framework that considers microbiomes as complex communities of interacting members to interpret and predict functional outcomes of fiber fermentation in future human intervention trials.

Although this study revealed ecological concepts to explain inter-individual variation in fiber fermentation of the human gut microbiota, we must acknowledge limitations in our ability to identify relevant players within trophic networks and ecological guilds using sequencing data from a human intervention study. Our analysis for the determination of CARGs was based on the correlation of compositional responses of the microbiota to arabinoxylan. Although this analysis identified clusters of species with traits to utilize arabinoxylan that are likely ecologically relevant, statistically significant correlations were also detected between CARGs, suggesting that trophic networks extend to the broader community. In addition, by being limited to correlations, our approach cannot identify causal links, and the focus on compositional shifts is unlikely to identify all members of trophic networks as not every species that contributes to the fermentation of a fiber becomes enriched [5]. There are, therefore, limitations in our ability to identify all relevant primary degraders, secondary fermenters, and metabolite utilizers, and more sensitive approaches such as stable isotope probing [63] or bio-orthogonal non-canonical amino acid tagging (BONCAT) [64] are required. Such studies could be complemented by co-culture experiments, such as those described by Ze et al. [17], to empirically test cross-feeding interactions and exert mechanisms by which gut bacteria collaborate to utilize specific fibers. The inclusion of such mechanistic information on trophic networks would likely improve the quality of models that predict the fermentation of dietary fiber and its metabolic consequences.

From an applied perspective, our findings have implications for the targeted use of arabinoxylan to modulate the gut microbiota for improved health. Probiotic treatments with *B*. *longum* strains have been shown to be health-promoting in a variety of contexts [65], including gastrointestinal [66, 67], immunological (e.g., anti-allergy and anti-inflammatory [68, 69]), and psychological (e.g., depression and anxiety [70, 71]) disorders. The specific enrichment of this species supports the use of arabinoxylan in synbiotic applications with *B*. *longum*. Another finding that warrants attention in the context of health is the increase in *P*. *copri*. Although the role of *P*. *copri* in human health remains unclear, with potential deleterious effects reported (e.g., enhanced rheumatoid arthritis susceptibility [72]) that are likely dependent on strain-level differences, dietary environments, and host predisposition [19, 73], this species was associated with improved glucose metabolism after whole grain barley treatment [8], and correlated with weight loss in volunteers that consumed diets high in whole grains [74, 75]. *Prevotella* is a genus that has been consistently negatively associated with an industrialized lifestyle [76, 77]. The reason for this reduction due to industrialization is unknown, but it has been speculated that reduced consumption of dietary fiber rich foods is responsible [78]. The increase of *P*. *copri* after supplementing through arabinoxylan supports this hypothesis, as arabinoxylan is a dominant fiber in whole grains, which are reduced in the westernized diet. The increased production of propionate would have implications for the treatment of obesity and related metabolic and immune alterations, as propionate administration has been shown to induce satiety [79], improve glucose homeostasis [80, 81], and suppress proinflammatory interleukin-8 levels [80] in humans. Overall, our findings suggest that arabinoxylan has prebiotic properties in that it promotes putatively health-related organisms and the production of propionate, making it a promising candidate for the prevention of obesity and associated pathologies, especially if its application is personalized.

## Conclusions

The findings of this study are relevant as individualized responses of the gut microbiota to dietary fiber provide a potential explanation for their inconsistent clinical effects in human intervention studies [6]. If metabolic functions relevant for the physiological effects of fiber (e.g., propionate) are individualized, then effects might not be detectable without stratifying the human population. This study further identified microbiota-related factors that can be used to predict arabinoxylan-induced propionate responses. Although significant MLR models were developed based on baseline microbiota profiles, which has practical advantages in personalizing intervention studies by the prediction of responses pre-treatment, the best models were obtained with compositional shifts, especially when features of the broader community (e.g., PCs) were considered. This finding serves as a proof-of-principle for the value of an ecological approach toward predictions of metabolic effects of fiber on the human gut microbiota. We acknowledge that the sample size of this exploratory study was too small to identify predictors of practical value that could be directly applied in independent studies. Larger studies are needed to develop robust machine learning algorithms—ideally informed through an ecological framework—that identify the exact factors that predict microbiota responses to dietary fiber.

## Methods

### Subjects

Male and pre-menopausal, non-pregnant or lactating female subjects aged 19 to 50 years with overweight or class-I obesity (BMI 25.0 to 34.9 kg/m^2^) and a stable body weight (± 3% for ≥ 1 month) who were otherwise healthy were recruited from the Edmonton area using campus-wide flyers, mailings to specific Listservs, local events, and word of mouth. Exclusion criteria included (1) history of gastrointestinal disorders or surgeries; (2) history of diabetes mellitus; (3) chronic use of anti-hypertensive, lipid-lowering, anti-diabetic, anti-inflammatory, or laxative medications; (4) antibiotic use three months prior to the study; (5) use of probiotic, prebiotic, omega-3 fatty acid, or herbal supplements; (6) intolerance to corn; (7) vegetarian; (8) smoking; (9) alcohol intake ≥ 7 drinks/week; and (10) > 3 h of moderate-vigorous exercise per week.

### Study design

This 6-week, parallel two-arm, exploratory randomized controlled trial (RCT) was prospectively registered on July 3, 2015 with ClinicalTrials.gov (NCT02322112) as part of a large parallel four-arm RCT that aimed to compare the effects of four structurally distinct fibers (i.e., arabinoxylan, acacia gum, resistant starch type-IV, and MCC) on the gut microbiota and human health, referred to as The Alberta FYBER (Feed Your Gut Bacteria morE fibeR) Study (for original registration we refer to [82]). In response to requests by reviewers of a grant application, which advised against including a premarket fiber ingredient in a larger human trial, the arabinoxylan arm was separated from the original RCT on October 26, 2016 and data from the 15 subjects that completed the protocol were analyzed independently. Study visits were conducted in accordance with the principles of the Declaration of Helsinki at the University of Alberta Human Nutrition Research Unit in Edmonton, Alberta, Canada between September 2015 and October 2016.

The study included five clinic visits (Fig. 1). During a 2-week screening/baseline period, potential subjects were pre-screened by telephone for initial eligibility and then attended a screening visit (visit 1) to confirm eligibility and receive study material (including fecal collection supplies) to be completed prior to the baseline visit (visit 2). During the baseline visit, eligible subjects were enrolled, stratified based on sex, and then randomly assigned to either the arabinoxylan arm or MCC arm. Random treatment allocation was accomplished using a computerized random number generator, in which two separate random allocation sequences (female and male sequence) were generated and concealed by a researcher not involved in subject allocation. Upon enrollment, subjects were then assigned to the next available randomization number by a study investigator blinded to these predetermined allocation sequences.

Thirty-eight subjects were enrolled in the study and instructed to consume their corresponding supplement for 6 weeks at a daily fiber dose of 25 g for females and 35 g for males, provided strictly as either arabinoxylan or MCC. Half daily doses were provided for the first 2 days of treatment (12.5 g for females and 17.5 g for males), as this was shown by pilot data to ease diet incorporation. After 1 week of treatment, subjects returned to provide a second fecal sample and to assess protocol adherence (visit 3), which was also assessed during their third week of treatment (visit 4). A final visit was required at endpoint (6 weeks of treatment) to provide the third and final fecal sample and to assess overall protocol adherence (visit 5).

### Treatments

The arabinoxylan used in this study was BIO-FIBER GUM and was provided by Agrifiber Holdings LLC (Illinois, USA) as a single batch. The long-chain arabinoxylan is an alkali-extract, soluble arabinoxylan isolated from corn bran that contained 81.0 ± 1.3% arabinoxylan. The arabinoxylan was further analyzed for its monosaccharide composition by their trimethylsilyl derivatives using gas chromatography coupled with mass spectroscopy (models 7890A and 5975C inert MSD with a Triple Axis detector, Agilent Technologies Inc., California, USA) as previously described [83]. The results showed that the corn bran arabinoxylan composes of 57.8% xylose and 32.5% arabinose (weight basis). As the backbone of arabinoxylan is comprised of linear xylose with arabinose forming branching points, the arabinose-to-xylose ratio is often used to estimate arabinoxylan branching density. The arabinoxylan used here had an arabinose-to-xylose ratio of 0.56, which is similar to that reported for alkali extracted corn arabinoxylans [42–44]. The arabinoxylan further contained 9.7% galactose, which is likely present in side chains as described for other corn arabinoxylans [42, 44]. The relatively high arabinose-to-xylose ratio and abundant galactose collectively suggest that the corn bran arabinoxylan is heavily branched with complex side chains, like the ones previously reported by Saulnier et al. [42], Rose et al. [43], and Rumpagaporn et al. [44]. The MCC used in this study was MICROCEL MC-12 and was provided by Blanver Farmoquimica LTDA (São Paulo, Brazil). The MCC is a large particle size (160-μm average), wood-derived cellulose fiber processed with a dilute-acid to remove amorphous regions leaving only recalcitrant crystalline regions. The MCC was subjected to in vitro fecal fermentations to confirm resistance to microbial fermentation and, therefore, selected as a non-fermentable control.

Both fibers were administered as powdered supplements and incorporated daily into the subjects’ preferred foods and drinks. The treatments were not identical in their appearance or physicochemical properties and, therefore, double-blinding was not possible. To achieve single-blinding, however, subjects were not informed of their fiber treatment, and weekly doses were provided in sealed opaque bags that contained individually packaged, ready-to-use fiber sachets. Subjects were instructed to return all provided sachets at their scheduled visits, where remaining fiber was weighed to assess protocol adherence.

### Baseline dietary intake and anthropometric assessment

Subjects were asked to maintain their habitual diet and physical activity level during the intervention study. Baseline dietary intake was assessed by the online Canadian Diet History Questionnaire II Past Month (C-DHQ II), a food frequency questionnaire adapted for the Canadian population from the validated US-DHQ II [84]. Subjects’ responses were analyzed using Diet*Calc software (Version 1.5.0) and the C-DHQ II-specific nutrient database; previously updated to include eight new food group variables representing Canada’s 2007 Food Guide serving-size-equivalents [85]. Prior to statistical analyses, C-DHQ II extracted data were assessed for extreme outliers using methods described by Kipnis and colleagues [86], and then calorie-adjusted using methods described by Willett and Stampfer [87].

Anthropometric measurements were also obtained at baseline and W6. Height and weight were measured, in light clothing, with empty pockets, and shoes removed, and used to calculate BMI. Waist circumference was measured using a Gulick II plus tape measure according to National Institutes of Health guidelines. Body fat percentage was estimated by bioelectrical impedance analysis (Tanita TBF-300A Body Composition Analyzer, Illinois, USA) using a proprietary equation.

### Assessment of stool consistency and bowel movement frequency

Self-reported stool consistency and bowel movement frequency were obtained at baseline and then at the end of each intervention week using a 5-point hedonic scale. For consistency, the scale was anchored by “hard or fragmented” (0) and “runny or watery” (4) with a score of two indicating normal or “smooth, soft, and formed” stool. For frequency, the scale was anchored by “every third day or less often” (0) and “three times a day or more often” (4) with a score of two indicating “once a day.” The area under the curve (AUC_BL–W6_) was then calculated using the linear trapezoidal method.

### Fecal sample collection and processing

Fecal samples were collected at baseline, W1, and W6 using stool collection kits consisting of a stool specimen container, an air-tight bag (Fisher, Canada), and a GasPak™ EZ Anaerobe Sachet (BD, Canada) to generate an anaerobic environment within the container. Samples were delivered to researchers within 4 h of defecation. Upon receipt, fecal samples were processed immediately in an anaerobic chamber (Bactron™, Shel Lab, Oregon, USA) with an environment consisting of 5% H_2_, 5% CO_2_, and 90% N_2_. Raw fecal material was aliquoted for pH and moisture content measurements, and also diluted 1:10 in molecular grade phosphate-buffered saline for DNA extractions and 1:5 5% phosphoric acid for SCFA quantification. Aliquots were stored at − 80 °C and kept frozen until further processing.

### Fecal pH, SCFA, and moisture content quantification

Raw fecal material was diluted 1:4 in distilled water to determine fecal pH using an Accumet AB150 pH meter (Fisher, Canada) as previously described [88, 89]. Quantification of fecal SCFAs was completed at the Agricultural, Food and Nutritional Science Chromatography Facility of the University of Alberta as previously described [90]. Briefly, 1:5 dilution of fecal samples homogenized in 5% phosphoric acid were thawed and centrifuged, then 1000 μl of supernatant was mixed with 200 μl of internal standard (4-methyl-valeric acid). Subsequently, 0.2 μl of the mixture was injected into a Bruker SCION 456 gas chromatograph (Bruker Corporation, Massachusetts, USA). SCFAs were separated on a Stabilwax-DA column (30 m × 0.53 mm inner diameter × 0.5 μm film thickness, Restek Corporation, Pennsylvania, USA) with a flame ionization detector, and quantified by calculating response factors for each SCFA relative to 4-methyl-valeric acid using injections of pure standards. Total SCFA concentrations were determined as the sum of acetate, propionate, and butyrate, while the relative percentage of each SCFA was determined by dividing these individual SCFAs by total SCFAs. Total branched short-chain fatty acid concentrations were determined as the sum of isobutyrate and isovalerate. Fecal moisture content was determined by drying raw fecal material overnight in an oven at 103 °C.

### DNA extraction, 16S ribosomal RNA (rRNA) gene amplicons sequencing, and data processing for microbiota analysis

Bacterial DNA was extracted from fecal homogenates in phosphate-buffered saline (1:10) using the QIAamp DNA Stool Mini Kit (QIAGEN, Hilden, Germany) as previously described [91]. The V5–V6 regions of the 16S rRNA gene were targeted for PCR amplification using primer pair 784F [5′-RGGATTAGATACCC-3′] and 1064R [5′-CGACRRCCATGCANCACCT-3′]. 16S rRNA gene amplicons were sequenced by 300 bp paired-end sequencing on the MiSeq platform at the University of Minnesota Genomics Center (Minnesota, USA), with all samples of this study being included in the same run.

Sequences were trimmed to 210 bases long using FASTX-Toolkit, and paired-end reads were merged with the merge-illumina-pairs pipeline as previously described [91]. Samples exceeding 16,000 reads were subsampled to 16,000 using USEARCH v8.1 [92]. Removal of chimeric reads and clustering of OTUs (at a 98% pairwise identity threshold) were conducted using USEARCH, resulting in an average of 10,763 ± 670 high-quality sequences per sample after quality control. Taxonomies from phylum to genus level were assigned using the entire sequence set by the Ribosomal Database Project Classifier [93]. OTUs were assigned taxonomy by using the Silva database (release 132 [94]), and sequence identity at species level was confirmed using 16S rRNA gene databases on EzBioCloud [95], IMG/MER [96], and NCBI [97] platforms.

Prior to ordination and statistical analysis, OTU count data were converted into relative abundance and also CLR transformed to correct for compositionality [98]. Considering all fecal samples, OTUs with an average relative abundance below 0.15% were removed. This approach resulted in exactly 100 OTUs (referred to as “all OTUs”), which were used in downstream analyses, accounting for 88.1% of the approximately 1 million-curated reads.

### Statistical analysis

All univariate analyses were performed by GraphPad Prism (v8.0.1; www.graphpad.com), while multivariate and regression model analyses were performed using R (v3.5.3; www.r-project.org) unless otherwise stated. The statistical analyses conducted are discussed in detail in the sections below.

### Bacterial community analysis

To explore the effect of fiber on the bacterial community, we assessed overall β-diversity, dissimilarity between and within individuals, and α-diversity. To assess overall β-diversity, Euclidean distance between bacterial communities was first calculated from CLR-transformed data of all OTUs and then visualized using non-metric multidimensional scaling (vegan [99] and ggplot2 [100] packages). Differences in the communities of arabinoxylan and MCC groups at specific time points were compared by PERMANOVA using the Adonis function in vegan [99]. Euclidean distances were used to calculate inter-subject (between subjects at the same time point) and intra-subject (within subjects, but at different time points) dissimilarities. Differences in inter-subject diversity were determined within each treatment group relative to baseline using GEE models (geepack package [101]) followed by Bonferroni correction. Differences of intra-subject dissimilarity between arabinoxylan and MCC were compared using Mann–Whitney tests. α-diversity (Shannon index) and bacterial richness (OTU numbers) were determined using rarefied OTU data with the vegan package [99].

### Fecal microbiota composition and SCFA analyses

Community membership of individual taxa was presented as relative abundance (mean ± SD), while CLR-transformed data were used for statistical analysis. Comparisons of phyla, families, genera, OTUs, and SCFAs between baseline and W6 were performed by Wilcoxon tests, while comparisons of shifts (i.e., ΔW6-baseline) between arabinoxylan and MCC were performed by Mann–Whitney tests. *P* values were adjusted by FDR and considered statistically significant when *q* values were less than 0.15. Differences at W1 and W6 in the effects of fiber on OTUs and SCFAs were determined using the Friedman’s test followed by a Dunn’s correction for multiple comparisons.

### CARG and network analyses

Potential syntrophic interactions between bacterial taxa in their response to arabinoxylan were assessed using co-occurrence network analysis [22]. To determine groups of interacting OTUs in their response to arabinoxylan (thus potential ecological guilds) [20], CARGs were determined from the top OTUs impacted by arabinoxylan consumption (ΔW6-baseline unadjusted *p* < 0.1; Wilcoxon test). Spearman’s correlation analysis was performed between the CLR-transformed shifts (ΔW6-baseline) in these OTUs to construct a correlation matrix using Spearman’s correlation coefficients, which was then converted into a distance matrix by (1—correlation coefficients) [22]. Next, hierarchical clustering was performed on the distance matrix to build a tree using the complete-linkage clustering algorithm (ComplexHeatmap package [102]) where branch lengths reflect the degree of association between OTUs (i.e., shorter branches indicate that OTU responses to arabinoxylan were more similar among individuals). Differences between distinct clusters of the Hierarchical tree, and thus individual CARGs, were determined by PERMANOVA using a cut-off of *p* < 0.05 [22]. In summary, OTUs within each CARG were observed to respond more similarly to arabinoxylan when compared to OTUs within another CARG, and these responses showed significant clustering, which suggests enhanced co-operative relationships between taxa of the same CARG during arabinoxylan degradation. Relative abundance of each CARG was calculated as the sum of the OTUs within each CARG prior to statistical analyses.

To visualize the interaction of OTUs within and between CARGs, a Spearman’s correlation network was calculated based on shifts in CLR-transformed abundance using permutation tests (1000×) by CoNet [103] as previously described [104]. To focus on the most robust interactions, only OTUs with Spearman’s rho values ≥ 0.5 or ≤ − 0.5 and FDR corrected *q* < 0.05 were visualized in the network using Cytoscape (v3.61; www.cytoscape.org).

### Differences in bacterial community composition and diet between W6-propionate responders and nonresponders

To identify factors that contribute to the variation between W6-propionate responders and nonresponders, PERMANOVA was performed on Euclidian distances based on the baseline and shifts of total OTUs, significant OTUs, CARGs, and baseline diet. The multivariate data of microbiota and diet were visualized on PCA biplots using factoextra [105] and FactoMineR [106] packages.

### Relationships between bacterial community and SCFA responses with microbiota and diet features

To explain the individualized response of the fecal microbiota to fiber, MLR analyses were employed using R. In order to perform the analysis, dimensionality of the microbiota and diet data were reduced by PCA into PC1, PC2, and PC3, which represents the largest proportion of the inter-individual variability and captures the most information on microbiota and dietary variation. Microbiota compositional and SCFA response variables were used as dependent variables. Baseline and shifts of PC variables, CARGs, OTUs, and diet data were used as predictors. Subset selection in regression was applied to choose the best combination of predictors using the sequential replacement algorithm (leaps package [107]). Therefore, each MLR model presented only contained the top one or two predictors that explained the response variable the best. Dietary and microbiota-related predictors were treated separately in different models, and total grains, whole grains, and total fiber intake were used as single dietary predictors. All models were adjusted by fiber dose/sex and *p* values were corrected by FDR with statistical significance considered at *q* < 0.05. To estimate the quality of each model in predicting the same dependent variable, AICc values were calculated using the AICcmodavg package [108]. AICc values were then converted to relative percentages by assigning the highest AICc value as 100%, and then remaining AICc values were calculated by $\frac{\mathrm{AICc}\ \mathrm{value}}{\mathrm{Highest}\ \mathrm{AICc}\ \mathrm{value}}\mathrm{x}\ 100$. Thus, lower AICc values indicate higher quality models. Residuals for all linear regression models were plotted to check for homogeneity of variance and normality.

## Supplementary information

**Additional file 1:** Figure S1. Flow chart summarizing subject flow through the study.

**Additional file 2:** Table S1. Subject characteristics at baseline. Table provides a list of the subject characteristics assessed at baseline, with subjects grouped based on their randomization to either the arabinoxylan or microcrystalline cellulose treatment arm. Data provided as mean ± SD or as a percentage

**Additional file 3: **Table S2. The relative abundance of bacterial taxa and co-abundance response groups (CARGs) affected by the dietary interventions as assessed by 16S rRNA gene amplicons sequencing. Table provides the relative abundances, relative changes, and *q* values of dominant bacterial taxa and CARGs affected by 6-week consumption of either arabinoxylan or microcrystalline cellulose (related to Fig. 2e). Data provided as mean ± SD

**Additional file 4:** Figure S2. Baseline fecal microbiota composition and diet showed no association with the individualized microbiota response to arabinoxylan. (A) Heatmap shows the associations between microbiota compositional shifts (ΔW6–BL; dependent variables; columns) and baseline microbiota profiles (predictors; rows). (B) Heatmap shows the association between microbiota compositional shifts (ΔW6–BL; dependent variables; columns) and the baseline diet variables (predictors; rows). For both A and B, cells represent individual multiple linear regression models (with FDR correction) that assess whether the predictors explain the individualized compositional shifts. Multivariate microbiota and diet data were simplified into principal component (PC) variables PC1, PC2, and PC3 prior to analysis. Each model contained the best one or two predictors of PCs (microbiota and diet), individual CARGs, or significant OTUs (predictors selected by stepwise regression), or either total grains, whole grains, or total fiber alone. All models were adjusted by fiber dose/sex. Colors from white to red indicate relative AICc (corrected Akaike information criterion) values calculated by (AICc value / Highest AICc value) x 100. Lower AICc values (red) indicate higher quality models. AX; arabinoxylan; BL, baseline; CARG, co-abundance response group; MCC, microcrystalline cellulose; OTU, operational taxonomic unit; W1, week 1; W6, week 6

**Additional file 5: **Table S3. Fecal pH, moisture content, and concentrations and percentages of fecal short-chain fatty acids. Table provides the actual values, relative changes, and *q* values of fecal pH, fecal moisture content, and fecal concentration and relative percentage of short-chain fatty acids measured as a response to 6-week arabinoxylan or microcrystalline cellulose supplementation (related to Fig. 5a)

**Additional file 6:** Figure S3. Effects of arabinoxylan and microcrystalline cellulose (MCC) on stool consistency and bowel movement (BM) frequency. (A) Stool consistency and (B) BM frequency changes induced by fiber supplementation. For A and B, line graphs show weekly self-reported stool consistency and BM frequency ratings, respectively; reported as mean ± SD. For A and B, bar graphs (insets) show area under the curve values (AUC_BL–W6_; mean ± SD). (C) Comparison between W6-responders (red) and W6-nonresponders (black) in stool consistency AUC_BL–W6_ and BM frequency AUC_BL–W6_. Data analyzed for (A,B) by generalized estimating equation models and for (A,B insets and C) by Mann-Whitney tests. BL, baseline; W1, week 1; W6, week 6

**Additional file 7:** Figure S4. Temporal propionate response to arabinoxylan supplementation showed no association with baseline diet. (A) Principal component analysis plot based on Euclidean distance comparing the baseline, calorie-adjusted intake of Canada’s 2007 Food Guide food group and macronutrient variables between W6-responders (red) and W6-nonresponders (black). Data were analyzed using PERMANOVA. (B) Comparison between W6-responders (red) and W6-nonresponders (black) in the calorie-adjusted intakes of single dietary factors (total grains, whole grains, total fiber, and arabinoxylan [AX] supplement) performed using Mann-Whitney tests. W1, week 1; W6, week 6

**Additional file 8:** Figure S5. Individualized acetate and butyrate response to arabinoxylan could be explained by baseline and shifts of the gut microbiota. Heatmap shows the associations between the individualized response of (A) acetate and (B) butyrate (ΔW6–BL; dependent variable; columns) and microbiota profiles (BL, ΔW1–BL, ΔW6–BL; predictors; rows). Cells represent individual multiple linear regression models (with FDR correction) that assess whether the predictors explain the individualized SCFA responses. Multivariate microbiota data were simplified into principal component (PC) variables PC1, PC2, and PC3 prior to analysis. Each model contained the best one or two predictors of PCs, individual CARGs, or significant OTUs selected by stepwise regression. All models were adjusted by fiber dose/sex. Colors from white to red indicate relative AICc (corrected Akaike information criterion) values calculated by (AICc value / Highest AICc value) x 100. Lower AICc values (red) indicate higher quality models. AX, arabinoxylan; BL, baseline; CARG, co-abundance response group; MCC, microcrystalline cellulose; OTU, operational taxonomic unit; W1, week 1; W6, week 6

**Additional file 9:** Figure S6. Individualized SCFA response to arabinoxylan could not be explained by baseline diet, stool consistency, or bowel movement (BM) frequency during treatment. Heatmap shows the associations between the individualized SCFA response (acetate, propionate, butyrate; dependent variable; columns) and either (A) baseline diet or (B) stool consistency and BM frequency (predictors; rows). For A and B, cells represent individual multiple linear regression models (with FDR correction) that assess whether the predictors explain the individualized SCFA responses. Multivariate diet data were simplified into principal component (PC) variables PC1, PC2, and PC3 prior to analysis. Each model contained either the calorie-adjusted intakes of total grains, whole grains, total fiber, or total supplemental fiber; stool consistency or BM frequency; or the best one or two diet PCs as the predictors (PCs selected by stepwise regression). All models were adjusted by fiber dose/sex. Colors from white to red indicate relative AICc (corrected Akaike information criterion) values calculated by (AICc value / Highest AICc value) x 100. Lower AICc values (red) indicate higher quality models. AX, arabinoxylan; BL, baseline; MCC, microcrystalline cellulose; SCFA, short-chain fatty acid; W6, week 6

**Additional file 10:** Table S4. Multiple linear regression analyses between arabinoxylan-induced fecal SCFA responses and bacterial features. Table provides the variables used for multiple linear regression analyses and corresponding results, which are presented as heatmaps in Fig. 7 and Fig. S5

**Additional file 11:** Table S5. Supporting metadata. Table provides relevant 16S rRNA gene amplicons sequencing metadata

## Abbreviations

AICc: Corrected Akaike information criterion
CLR: Centered log-ratio
CARG: Co-abundance response group
FDR: Benjamini-Hochberg’s false discovery rate
MCC: Microcrystalline cellulose
MLR: Multiple linear regression
OTU: Operational taxonomic unit
PCA: Principal component analysis
PC: Principal component
PERMANOVA: Permutational multivariate analysis of variance
SCFA: Short-chain fatty acid
W1: Week 1
W6: Week 6
