# Supplementary figures and images for: Gut microbiota modulation with long-chain corn bran arabinoxylan in adults with overweight and obesity is linked to an individualized temporal increase in fecal propionate
Source: Microbiome. 2020 Aug 19;8:118. doi: 10.1186/s40168-020-00887-w (PMC7439537; doi:10.1186/s40168-020-00887-w)

**Additional file 1: Figure S1.**

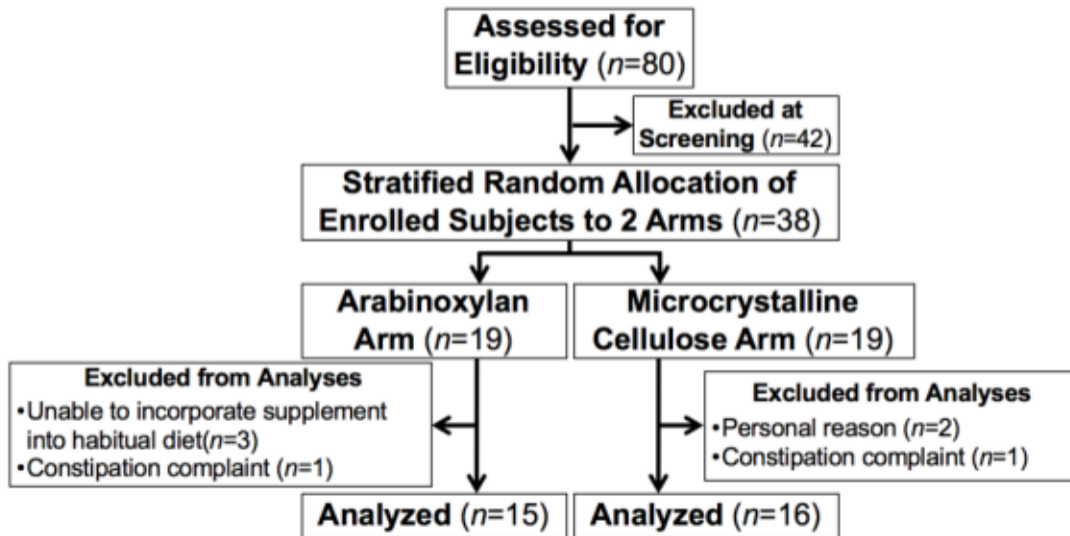

Supplement: Supplementary file 2 — Additional file 1: Figure S1. Flow chart summarizing subject flow through the study. [file 40168_2020_887_MOESM1_ESM.pdf]

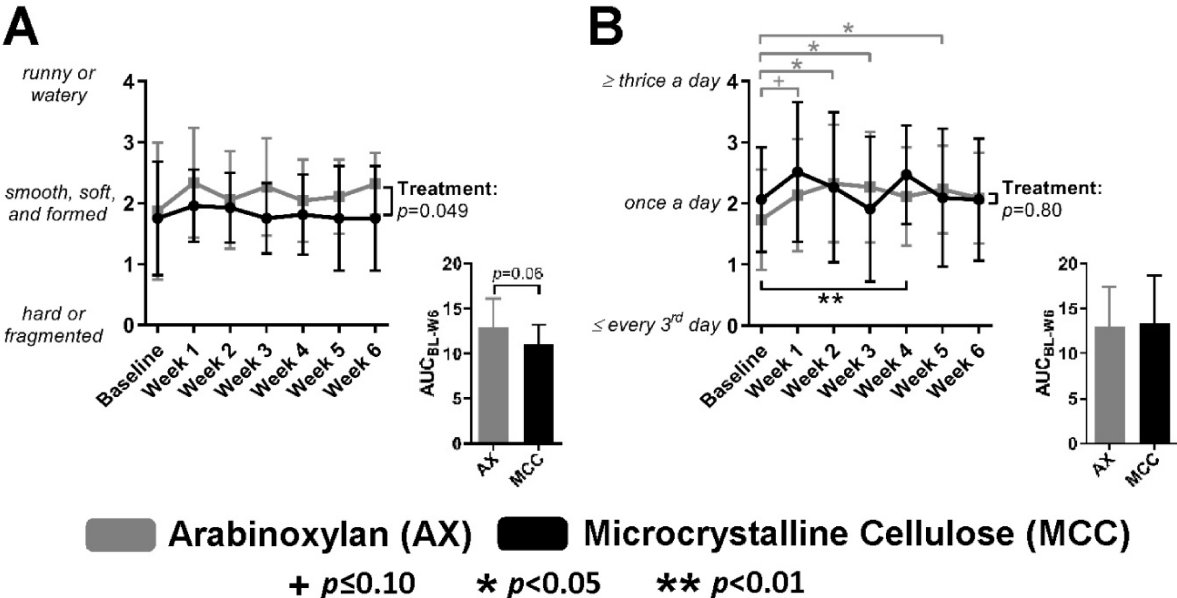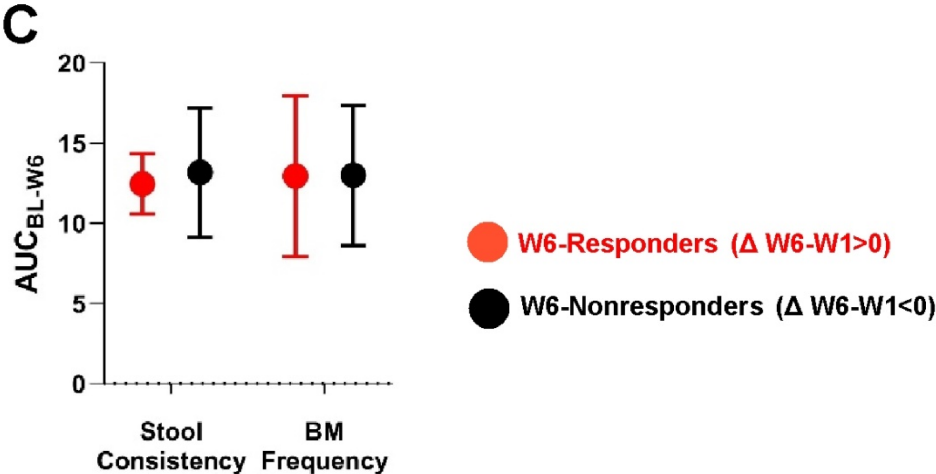

Supplement: Supplementary file 7 — Additional file 6: Figure S3. Effects of arabinoxylan and microcrystalline cellulose (MCC) on stool consistency and bowel movement (BM) frequency. (A) Stool consistency and (B) BM frequency changes induced by fiber supplementation. For A and B, line graphs show weekly self-reported stool consistency and BM frequency ratings, respectively; reported as mean ± SD. For A and B, bar graphs (insets) show area under the curve values (AUCBL–W6; mean ± SD). (C) Comparison between W6-responders (red) and W6-nonresponders (black) in stool consistency AUCBL–W6 and BM frequency AUCBL–W6. Data analyzed for (A,B) by generalized estimating equation models and for (A,B insets and C) by Mann-Whitney tests. BL, baseline; W1, week 1; W6, week 6 [file 40168_2020_887_MOESM6_ESM.pdf]

Additional file 7: Figure S4.

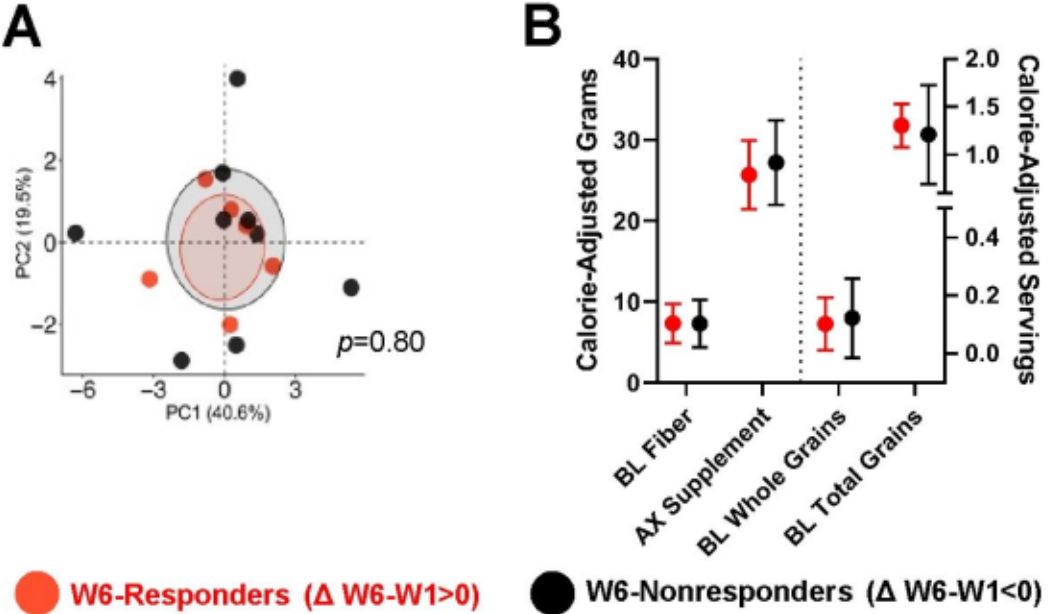

Supplement: Supplementary file 8 — Additional file 7: Figure S4. Temporal propionate response to arabinoxylan supplementation showed no association with baseline diet. (A) Principal component analysis plot based on Euclidean distance comparing the baseline, calorie-adjusted intake of Canada’s 2007 Food Guide food group and macronutrient variables between W6-responders (red) and W6-nonresponders (black). Data were analyzed using PERMANOVA. (B) Comparison between W6-responders (red) and W6-nonresponders (black) in the calorie-adjusted intakes of single dietary factors (total grains, whole grains, total fiber, and arabinoxylan [AX] supplement) performed using Mann-Whitney tests. W1, week 1; W6, week 6 [file 40168_2020_887_MOESM7_ESM.pdf]

**A**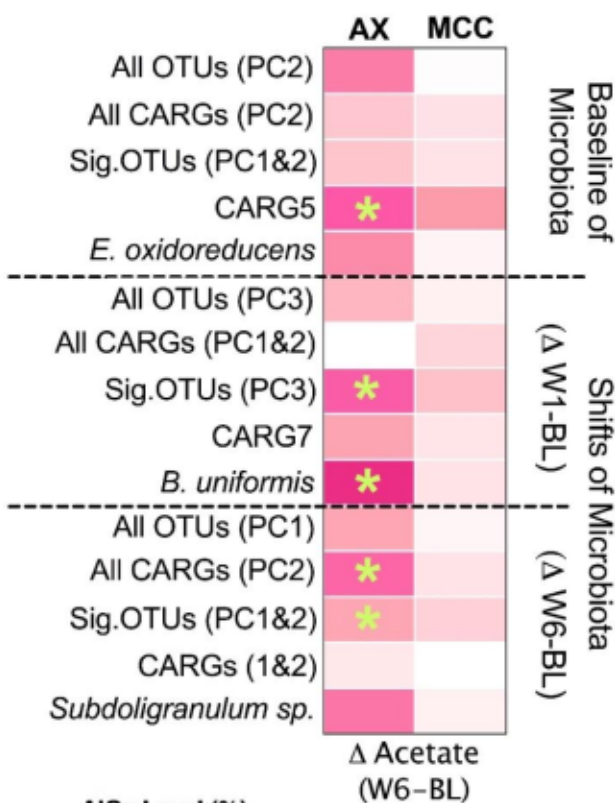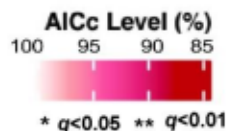**B**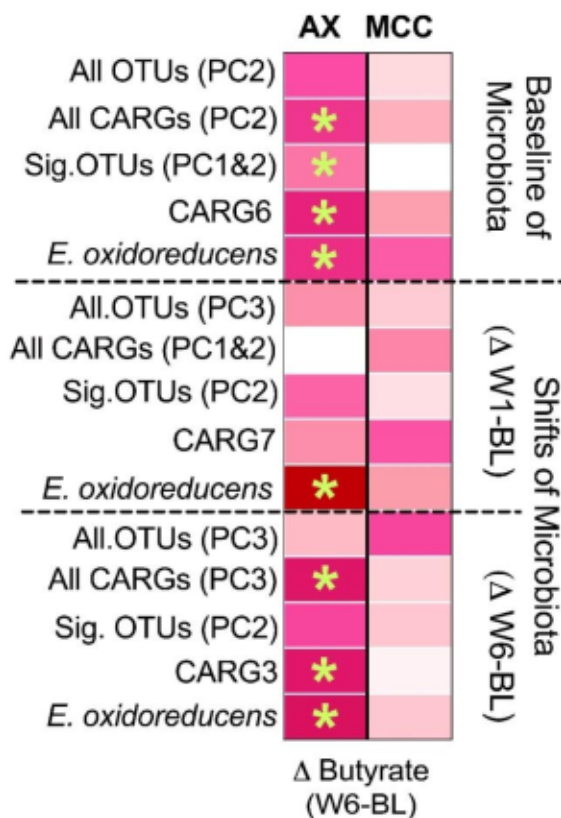

Supplement: Supplementary file 9 — Additional file 8: Figure S5. Individualized acetate and butyrate response to arabinoxylan could be explained by baseline and shifts of the gut microbiota. Heatmap shows the associations between the individualized response of (A) acetate and (B) butyrate (ΔW6–BL; dependent variable; columns) and microbiota profiles (BL, ΔW1–BL, ΔW6–BL; predictors; rows). Cells represent individual multiple linear regression models (with FDR correction) that assess whether the predictors explain the individualized SCFA responses. Multivariate microbiota data were simplified into principal component (PC) variables PC1, PC2, and PC3 prior to analysis. Each model contained the best one or two predictors of PCs, individual CARGs, or significant OTUs selected by stepwise regression. All models were adjusted by fiber dose/sex. Colors from white to red indicate relative AICc (corrected Akaike information criterion) values calculated by (AICc value / Highest AICc value) x 100. Lower AICc values (red) indicate higher quality models. AX, arabinoxylan; BL, baseline; CARG, co-abundance response group; MCC, microcrystalline cellulose; OTU, operational taxonomic unit; W1, week 1; W6, week 6 [file 40168_2020_887_MOESM8_ESM.pdf]

**A**

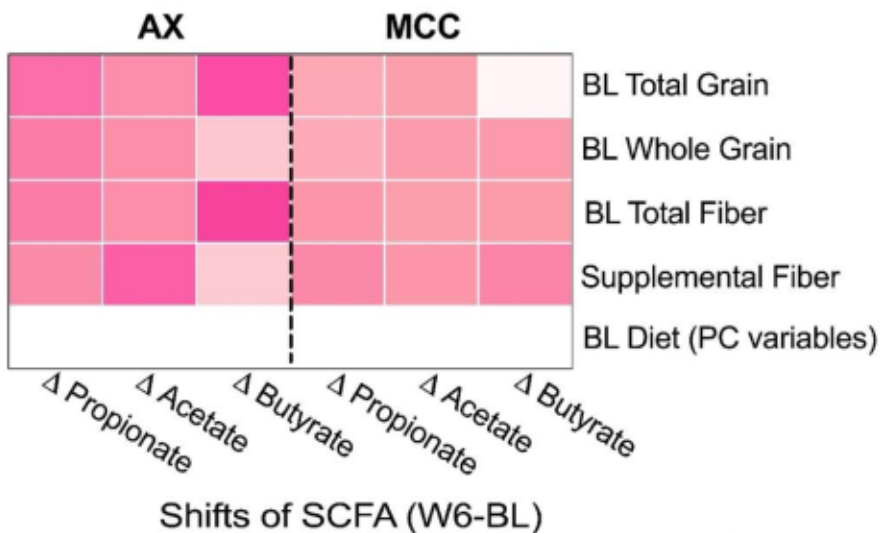

**B**

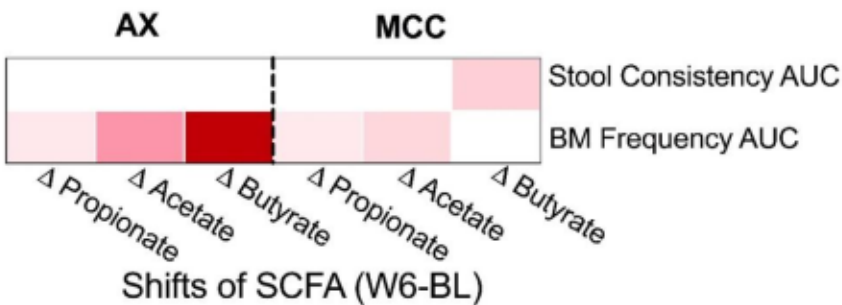

Supplement: Supplementary file 10 — Additional file 9: Figure S6. Individualized SCFA response to arabinoxylan could not be explained by baseline diet, stool consistency, or bowel movement (BM) frequency during treatment. Heatmap shows the associations between the individualized SCFA response (acetate, propionate, butyrate; dependent variable; columns) and either (A) baseline diet or (B) stool consistency and BM frequency (predictors; rows). For A and B, cells represent individual multiple linear regression models (with FDR correction) that assess whether the predictors explain the individualized SCFA responses. Multivariate diet data were simplified into principal component (PC) variables PC1, PC2, and PC3 prior to analysis. Each model contained either the calorie-adjusted intakes of total grains, whole grains, total fiber, or total supplemental fiber; stool consistency or BM frequency; or the best one or two diet PCs as the predictors (PCs selected by stepwise regression). All models were adjusted by fiber dose/sex. Colors from white to red indicate relative AICc (corrected Akaike information criterion) values calculated by (AICc value / Highest AICc value) x 100. Lower AICc values (red) indicate higher quality models. AX, arabinoxylan; BL, baseline; MCC, microcrystalline cellulose; SCFA, short-chain fatty acid; W6, week 6 [file 40168_2020_887_MOESM9_ESM.pdf]
