# Supplementary material for: Gut microbiota modulation with long-chain corn bran arabinoxylan in adults with overweight and obesity is linked to an individualized temporal increase in fecal propionate
Source: Microbiome. 2020 Aug 19;8:118. doi: 10.1186/s40168-020-00887-w (PMC7439537; doi:10.1186/s40168-020-00887-w)
Supplement: Supplementary file 3 — Additional file 2: Table S1. Subject characteristics at baseline. Table provides a list of the subject characteristics assessed at baseline, with subjects grouped based on their randomization to either the arabinoxylan or microcrystalline cellulose treatment arm. Data provided as mean ± SD or as a percentage [file 40168_2020_887_MOESM2_ESM.pdf]

Additional file 2: Table S1. Subject Characteristics at Baseline[1].

	Arabinoxylan	Microcrystalline Cellulose	Between Group *p* value [2]
Number	15	16	
Age (y)	33.7 ± 9.7	32.1 ± 7.4	0.91
Sex (female/male)	10/5	11/5	
Height (cm)	171.5 ± 8.4	168.8 ± 7.6	0.25
Weight (kg)	84.8 ± 12.3	81.9 ± 10.5	0.40
BMI (kg/m^2)	28.7 ± 2.7	28.7 ± 2.0	0.99
Waist circumference (cm)	95.7 ± 8.8	92.9 ± 6.0	0.30
Percent body fat (%)	33.0 ± 9.3	32.0 ± 7.3	0.63
Females	36.4 ± 2.9	38.0 ± 6.1	0.20
Males	22.5 ± 3.6	23.0 ± 5.3	0.84
Ethnicity (%)			0.56
White	60.0	43.8	
Asian	20.0	31.3	
Black	13.3	6.3	
Other	6.7	18.8	
Employment (%)			0.40
Student	26.7	50.0	
Employed	66.7	43.8	
Unemployed	6.7	6.3	

[1] Data presented as mean ± standard deviation or as a percentage.

[2] Continuous variables were analyzed by Mann-Whitney test, and count variables were analyzed by Chi-Squared test. BMI, body mass index.
