# Supplementary material for: Gut microbiota modulation with long-chain corn bran arabinoxylan in adults with overweight and obesity is linked to an individualized temporal increase in fecal propionate
Source: Microbiome. 2020 Aug 19;8:118. doi: 10.1186/s40168-020-00887-w (PMC7439537; doi:10.1186/s40168-020-00887-w)
Supplement: Supplementary file 4 — Additional file 3: Table S2. The relative abundance of bacterial taxa and co-abundance response groups (CARGs) affected by the dietary interventions as assessed by 16S rRNA gene amplicons sequencing. Table provides the relative abundances, relative changes, and q values of dominant bacterial taxa and CARGs affected by 6-week consumption of either arabinoxylan or microcrystalline cellulose (related to Fig. 2e). Data provided as mean ± SD [file 40168_2020_887_MOESM3_ESM.pdf]

Additional file 3: Table S2. Abundance of Bacterial Taxa and CARGs Affected by the Dietary Interventions as Assessed by 16S rRNA Gene Amplicons Sequencing[1].

	Proportion of bacterial taxa expressed in relative abundance (Mean ± Standard Deviation)								
	Arabinoxylan (*n*=15)				Microcrystalline Cellulose (*n*=16)				Between Group
Taxonomic Group	Baseline	Week 6	Within Group *p* value (adj.)	Δ Taxa	Baseline	Week 6	Within Group *p* value (adj.)	Δ Taxa	*p* value (adj.)
**Phyla**									
Firmicutes	65.76 ± 7.49	59.80 ± 5.76	0.761(0.903)	-5.95 ± 8.59	64.2 ± 10.0	65.8 ± 9.05	0.143(0.457)	1.68 ± 6.82	0.281(0.856)
Bacteroidetes	17.19 ± 6.87	21.90 ± 7.44	0.021(0.215)	-4.70 ± 5.56	19.9 ± 6.57	21.8 ± 7.05	0.231(0.505)	1.91 ± 8.84	0.519(0.931)
Actinobacteria	11.41 ± 8.74	13.17 ± 8.60	0.072(0.300)	1.76 ± 5.01	7.67 ± 4.94	6.6 ± 4.45	0.403(0.743)	-1.12 ± 4.26	0.065(0.416)
**Family**									
Erysipelotrichaceae	7.27 ± 6.25	3.82 ± 3.52	**0.0001(0.004)**	-3.45 ± 3.98	5.56 ± 5.09	5.40 ± 4.05	0.820(0.957)	-0.17 ± 4.22	**0.015(0.149)**
Lachnospiraceae	30.66 ± 6.60	30.20 ± 4.33	0.359(0.567)	-0.46 ± 7.86	26.7 ± 10.1	30.4 ± 9.46	**0.002(0.117)**	3.65 ± 7.54	0.188(0.731)
Bifidobacteriaceae	5.41 ± 5.37	8.50 ± 7.15	**0.0026(0.036)**	3.08 ± 4.20	3.68 ± 4.16	3.3 ± 4.26	0.668(0.882)	-0.38 ± 2.86	**0.017(0.149)**
**Genera**									
Blautia	3.03 ± 2.18	4.28 ± 2.21	0.025(0.223)	1.24 ± 2.19	3.11 ± 3.1	2.40 ± 2.02	0.463(0.806)	-0.64 ± 1.64	**0.010(0.149)**
Clostridium XVIII	1.10 ± 1.14	0.40 ± 0.44	**0.0003(0.0007)**	-0.76 ± 0.85	0.78 ±1.21	1.37 ± 2.67	0.705(0.898)	0.58 ± 1.55	**0.011(0.149)**
Lachnospiracea incertae sedis	7.13 ± 4.42	5.24 ± 3.72	0.120(0.324)	-1.89 ± 5.59	5.57 ± 4.04	7.95 ± 6.90	0.044(0.310)	2.38 ± 5.15	**0.017(0.149)**
Ruminococcus2	2.10 ± 1.63	1.42 ± 1.32	0.041(0.258)	-0.67 ± 1.36	1.41 ± 0.73	2.16 ± 1.53	0.028(0.289)	0.74 ± 1.17	**0.005(0.149)**
Prevotella	0.99 ± 1.92	3.75 ± 7.66	**0.0001(0.006)**	2.75 ± 6.19	4.25 ± 8.49	5.10 ± 9.13	0.010(0.229)	0.85 ± 4.97	0.740(0.959)
Bifidobacterium	5.41 ± 5.37	8.50 ± 7.15	**0.002(0.003)**	3.08 ± 4.20	3.68 ± 4.17	3.29 ± 4.26	0.632(0.867)	-0.38 ± 2.86	**0.017(0.149)**
Megamonas	1.44 ± 4.21	2.21 ± 5.37	0.151(0.353)	0.76 ± 1.60	0.14 ± 0.51	0.31 ± 0.87	0.013(0.229)	0.17 ± 0.48	0.598(0.931)
Parasutterella	0.58 ± 0.88	0.54 ± 1.14	0.389(0.567)	-0.04 ± 0.55	0.40 ±0.80	0.66 ± 1.00	**0.003(0.117)**	0.25 ± 0.53	**0.011(0.149)**
**Operational Taxonomic Units *(OTU Number, closest hit in database, % identity)***									
OTU6, *Prevotella copri*, 99%	0.95 ± 1.84	3.62 ± 7.41	**0.0001(0.006)**	2.67 ± 6.08	3.61± 7.53	4.41 ± 8.02	0.175(0.833)	0.79 ± 5.24	0.1194(0.398)
OTU79, *Muribaculaceae*	0.07 ± 0.21	0.14 ± 0.38	**0.0001(0.006)**	0.07 ± 0.21	0.50 ± 1.84	0.11 ± 0.45	0.596(0.932)	-0.38 ± 1.39	**0.0003(0.036)**
OTU11, *Subdoligranulum* sp.	1.26 ± 1.67	2.41 ± 3.27	**0.0020(0.042)**	1.14 ± 1.89	1.78 ± 1.89	1.38 ± 1.39	0.781(0.977)	-0.39 ± 1.24	0.1880(0.480)
OTU21, *Faecalibacillus intestinalis/faecis*, 100%	1.74 ± 1.25	0.81 ± 0.69	**0.0026(0.042)**	-0.92 ± 1.13	1.71 ± 1.44	1.95 ± 2.52	0.433(0.833)	0.23 ± 1.90	**0.0105(0.105)**
OTU38, *Phascolarctobacterium succinatutens*, 100%	0.39 ± 0.95	0.77 ± 1.68	**0.0043(0.042)**	0.38 ± 0.80	0.25 ±1.00	0.44 ± 1.78	0.322(0.671)	0.19 ± 0.78	**0.0063(0.105)**
OTU4, *Bifidobacterium longum*, 100%	3.53 ± 4.35	7.04 ± 6.76	**0.0020(0.042)**	3.51 ± 4.51	1.55 ± 2.24	1.64 ± 3.96	0.375(0.833)	0.09 ± 1.99	**0.0105(0.105)**
OTU46, *Clostridium leptum*, 100%	0.31 ± 0.77	0.56 ± 1.31	**0.0043(0.042)**	0.25 ± 0.58	0.08 ± 0.08	0.56 ± 1.35	0.403(0.833)	0.47 ± 1.35	**0.0105(0.105)**
OTU53, *Bacteroides plebeius,* 98.76%	0.20 ± 0.55	0.56 ± 1.58	**0.0043(0.042)**	0.35 ± 1.20	0.72 ± 2.66	1.06 ± 2.97	0.297(0.833)	0.34 ± 0.74	0.4700(0.723)
OTU56, *Bacteroides* sp.	0.15 ± 0.66	0.84 ± 3.04	**0.0043(0.042)**	0.68 ± 2.44	BDL	BDL	BDL	BDL	BDL
OTU85, *Blautia obeum*, 100%	0.43 ± 0.53	1.33 ± 1.30	**0.0034(0.042)**	0.89 ± 1.31	0.55 ± 0.55	0.43 ± 0.37	0.375(0.833)	-0.11 ± 0.46	**0.0041(0.105)**
OTU32, *Mollicutes*	0.11 ± 0.43	0.35 ± 1.34	**0.0084(0.076)**	0.23 ± 0.91	1.59 ± 3.91	0.80 ± 1.95	0.073(0.671)	-0.79 ± 2.08	**0.0090(0.105)**

Additional file 3: Table S2. *Continued*

Taxonomic Group	Arabinoxylan (*n*=15) Baseline	Week 6	Within Group *p* value (adj.)	Δ Taxa	Microcrystalline Cellulose (*n*=16) Baseline	Week 6	Within Group *p* value (adj.)	Δ Taxa	Between Group *p* value (adj.)
OTU41, *Eubacterium oxidoreducens*, 99%	0.96 ± 1.08	0.42 ± 0.48	**0.0151(0.125)**	-0.53 ± 0.70	1.07 ±0.98	0.85 ± 0.80	0.705(0.977)	-0.22 ± 0.71	**0.0009(0.105)**
OTU26, *Bacteroides ovatus*, 100%	0.41 ± 0.91	0.98 ± 1.16	**0.0181(0.129)**	0.57 ± 1.26	0.26 ± 0.28	0.54 ± 0.69	0.024(0.356)	0.31 ± 0.55	0.3182(0.578)
OTU5, *Ruminococcus bromii*, 100%	1.25 ± 1.56	0.87 ± 1.31	**0.0181(0.129)**	-0.38 ± 0.90	2.98 ± 2.94	2.78 ± 3.30	0.743(0.977)	-0.19 ± 1.74	0.0710(0.326)
OTU7, *Bacteroides uniformis*, 100%	2.38 ± 1.99	1.68 ± 2.65	**0.0021(0.143)**	-0.70 ± 2.23	1.73 ± 2.22	1.55 ± 1.60	0.820(0.977)	-0.17 ± 1.12	0.0780(0.326)
OTU65, *Ruminococcus lactaris*, 100%	0.37 ± 0.65	0.09 ± 0.29	0.0353(0.185)	-0.27 ± 0.62	0.24 ± 0.32	0.34 ± 0.41	0.073(0.671)	0.11 ± 0.25	**0.0072(0.105)**
OTU432, *Bacteroides cellulosilyticus*, 98.76%	0.16 ± 0.44	1.09 ± 2.99	0.0255(0.150)	0.93 ± 2.56	0.14 ± 0.27	0.06 ± 0.13	0.252(0.833)	-0.08 ± 0.24	**0.0105(0.105)**
OTU31, *Blautia faecis*, 100%	1.45 ± 1.63	1.64 ± 1.06	0.1876(0.360)	0.19 ± 1.47	0.94 ± 0.65	0.58 ± 0.45	0.015(0.356)	-0.36 ± 0.46	**0.0105(0.105)**
OTU10, *Holdemanella biformis*, 99.58%	4.52 ± 5.95	2.31 ± 3.36	0.1069(0.248)	-2.20 ± 3.25	1.98 ± 2.81	1.65 ± 2.48	0.018(0.356)	-0.33 ± 0.72	0.7700(0.906)
OTU116, *Eubacterium ramulus*, 100%	0.22 ± 0.23	0.21 ± 0.29	0.6787(0.780)	-0.01± 0.23	0.11 ± 0.17	0.19 ± 0.18	0.018(0.356)	0.08 ± 0.15	0.0405(0.155)
OTU47, *Bacteroides xylanisolvens*, 100%	0.26 ± 0.36	0.58 ± 1.01	0.4887(0.634)	0.31 ± 0.95	0.33 ± 0.53	0.88 ± 1.53	0.007(0.356)	0.55 ± 1.09	0.0855(0.329)
OTU54, *Parasutterella excrementihominis*, 100%	0.58 ± 0.89	0.54 ± 1.15	0.3591(0.561)	-0.04 ± 0.55	0.38 ± 0.78	0.64 ± 0.97	0.013(0.356)	0.26 ± 0.54	0.0170(0.155)
**Co-Abundance Response Groups (CARGs) (Sum of relative abundance of OTUs within each CARG)**									
CARG1	5.97 ± 4.97	12.9 ± 11.02	**0.0034**	6.98 ± 8.76	4.86 ± 3.70	4.98 ± 4.72	0.97	0.12 ± 2.64	**0.0072**
CARG2	2.56 ± 1.21	6.09 ± 6.09	**0.0084**	3.53 ± 5.30	2.92 ± 2.51	3.22 ± 2.43	0.46	0.29 ± 1.19	**0.0170**
CARG3	1.21 ± 1.58	2.48 ± 3.58	**0.0200**	1.27 ± 2.83	2.37 ± 2.51	2.86 ± 2.94	0.07	0.48 ± 1.65	0.4600
CARG4	2.90 ± 4.09	3.94 ± 4.61	0.1600	1.04 ± 2.57	3.29 ± 4.20	2.85 ± 2.69	0.85	-0.43 ± 2.95	0.3100
CARG5	1.94 ± 1.90	1.78 ± 1.42	0.7100	-0.15 ± 1.82	1.66 ± 1.64	2.43 ± 2.86	0.14	0.77 ± 2.27	0.2400
CARG6	4.63 ± 3.59	6.34 ± 8.30	**0.0200**	1.70 ± 6.83	7.50 ± 7.18	9.14 ± 7.84	0.43	1.63 ± 5.76	0.4900
CARG7	13.82 ± 8.19	8.21 ± 6.49	**0.0200**	-5.60 ± 8.69	11.7 ± 5.93	14.4 ± 7.73	0.25	2.33 ± 6.20	**0.0063**

[1] Statistical significance of within-group shifts (Δ week 6-baseline) were determined by Wilcoxon tests, while between-group shifts (Δ arabinoxylan vs. Δ microcrystalline cellulose) were determined by Mann-Whitney tests. Data are presented as relative abundance, and were centered log-ratio (CLR) transformed prior to the statistical analyses. *p* values were adjusted by FDR, whereas FDR significance was set at $q<0.15$. BDL, below detection limit; Δ Taxa, absolute change in relative abundance from baseline to week 6.
