# Supplementary material for: Gut microbiota modulation with long-chain corn bran arabinoxylan in adults with overweight and obesity is linked to an individualized temporal increase in fecal propionate
Source: Microbiome. 2020 Aug 19;8:118. doi: 10.1186/s40168-020-00887-w (PMC7439537; doi:10.1186/s40168-020-00887-w)

Additional file 4: Figure S2.

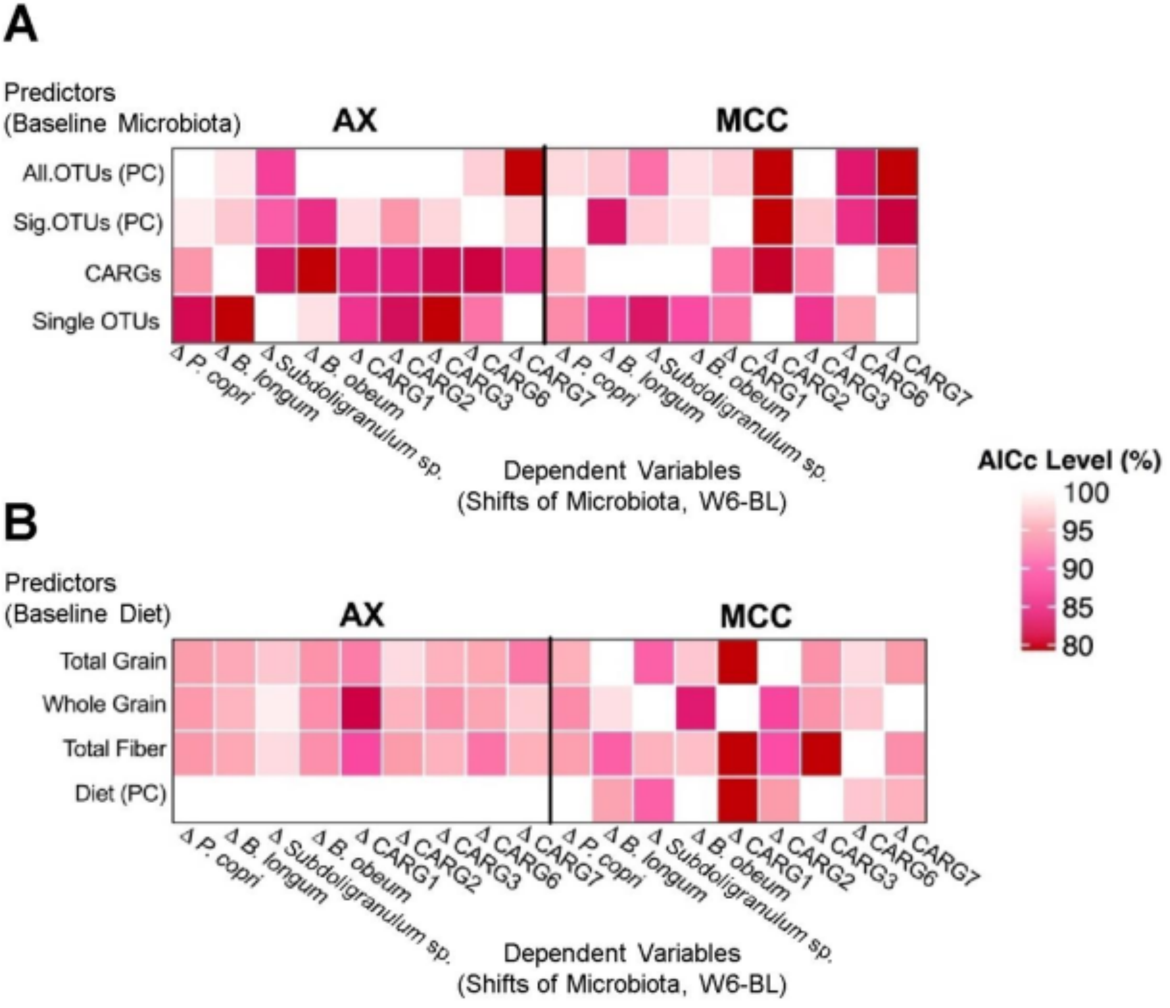

Supplement: Supplementary file 5 — Additional file 4: Figure S2. Baseline fecal microbiota composition and diet showed no association with the individualized microbiota response to arabinoxylan. (A) Heatmap shows the associations between microbiota compositional shifts (ΔW6–BL; dependent variables; columns) and baseline microbiota profiles (predictors; rows). (B) Heatmap shows the association between microbiota compositional shifts (ΔW6–BL; dependent variables; columns) and the baseline diet variables (predictors; rows). For both A and B, cells represent individual multiple linear regression models (with FDR correction) that assess whether the predictors explain the individualized compositional shifts. Multivariate microbiota and diet data were simplified into principal component (PC) variables PC1, PC2, and PC3 prior to analysis. Each model contained the best one or two predictors of PCs (microbiota and diet), individual CARGs, or significant OTUs (predictors selected by stepwise regression), or either total grains, whole grains, or total fiber alone. All models were adjusted by fiber dose/sex. Colors from white to red indicate relative AICc (corrected Akaike information criterion) values calculated by (AICc value / Highest AICc value) x 100. Lower AICc values (red) indicate higher quality models. AX; arabinoxylan; BL, baseline; CARG, co-abundance response group; MCC, microcrystalline cellulose; OTU, operational taxonomic unit; W1, week 1; W6, week 6 [file 40168_2020_887_MOESM4_ESM.pdf]
