# Supplementary material for: Gut microbiota modulation with long-chain corn bran arabinoxylan in adults with overweight and obesity is linked to an individualized temporal increase in fecal propionate
Source: Microbiome. 2020 Aug 19;8:118. doi: 10.1186/s40168-020-00887-w (PMC7439537; doi:10.1186/s40168-020-00887-w)
Supplement: Supplementary file 6 — Additional file 5: Table S3. Fecal pH, moisture content, and concentrations and percentages of fecal short-chain fatty acids. Table provides the actual values, relative changes, and q values of fecal pH, fecal moisture content, and fecal concentration and relative percentage of short-chain fatty acids measured as a response to 6-week arabinoxylan or microcrystalline cellulose supplementation (related to Fig. 5a) [file 40168_2020_887_MOESM5_ESM.pdf]

Additional file 5: Table S3. Fecal pH, Moisture Content, and Concentrations and Percentages of Fecal Short-Chain Fatty Acids[1].

	Fecal pH, Moisture Content, and Short-Chain Fatty Acids (Mean ± Standard Deviation)								
	Arabinoxylan (*n*=15)				Microcrystalline Cellulose (*n*=16)				Between Group *p* value (adj.)
	Baseline	Week 6	Within Group *p* value (adj.)	Δ SCFA	Baseline	Week 6	Within Group *p* value (adj.)	Δ SCFA	
**Fecal pH**	6.8 ± 0.5	6.7 ± 0.5	0.57(1.00)	-0.1 ± 0.6	6.8 ± 0.6	7.0 ± 0.6	0.18(0.76)	0.3 ± 0.8	0.095(0.31)
**Fecal moisture content (%)**	71.7 ± 7.3	72.4 ± 7.0	0.80(1.00)	0.7 ± 6.7	72.2 ± 8.7	69.9 ± 7.2	0.40(0.76)	-2.3 ± 11.2	0.52(0.70)
**Fecal Concentration (µmol/g)**									
Total SCFAs	145.3 ± 99.0	151.4 ± 80.7	0.59(1.00)	6.2 ± 65.2	145.3 ± 111.9	126.2 ± 55.2	0.59(0.76)	-19.0 ± 96.9	0.56(0.70)
Acetate	90.2 ± 56.2	94.8 ± 50.9	0.99(1.00)	4.6 ± 37.0	91.4 ± 63.6	80.8 ± 30.3	0.32(0.76)	-10.6 ± 56.6	0.46(0.70)
Propionate	31.2 ± 28.6	39.0 ± 26.0	0.15(0.53)	7.8 ± 21.1	31.7 ± 32.4	24.0 ± 13.5	0.40(0.76)	-7.7 ± 24.8	0.11(0.31)
Butyrate	23.8 ± 18.4	17.6 ± 10.3	0.48(1.00)	-6.3 ± 16.7	22.3 ± 19.3	21.4 ± 16.9	0.93(0.94)	-0.8 ± 21.9	0.59(0.70)
Valerate	3.1 ± 1.6	3.1 ± 1.7	0.93(1.00)	-0.004 ± 1.1	3.5 ± 2.7	3.1 ± 1.6	0.59(0.76)	-0.3 ± 2.4	0.76(0.77)
Total BCFAs	7.8 ± 3.6	8.4 ± 4.6	0.71(1.00)	0.6 ± 3.8	7.4 ± 3.6	6.4 ± 2.5	0.43(0.76)	-1.1 ± 4.8	0.39(0.70)
Isobutyrate	3.1 ± 1.1	3.4 ± 1.6	0.52(1.00)	0.3 ± 1.5	2.9 ± 1.3	2.6 ± 0.9	0.49(0.76)	-0.2 ± 1.8	0.26(0.61)
Isovalerate	4.7 ± 2.5	5.0 ± 3.0	0.97(1.00)	0.3 ± 2.4	4.6 ± 2.5	3.7 ± 1.7	0.29(0.76)	-0.8 ± 3.2	0.46(0.70)
**Percentage of Total SCFAs (%)**									
Acetate	62.7 ± 6.4	62.7 ± 6.7	0.97(1.00)	-0.04 ± 6.9	66.1 ± 6.1	66.6 ± 9.2	0.78(0.92)	1.0 ± 8.3	0.66(0.72)
Propionate	20.7 ± 6.3	25.0 ± 6.8	0.04(0.19)	4.3 ± 7.8	18.9 ± 4.2	17.8 ± 5.6	0.23(0.76)	-1.4 ± 4.5	**0.01(0.07)**
Butyrate	16.6 ± 5.0	12.3 ± 5.2	**0.018(0.13)**	-4.3 ± 6.4	15.0 ± 4.1	15.9 ± 7.1	0.89(0.94)	0.4 ± 7.0	0.08(0.31)
**Propionate-to-Butyrate Ratio**	1.4 ± 0.5	3.3 ± 4.6	**0.004(0.06)**	2.0 ± 4.7	1.4 ± 0.6	1.4 ± 0.7	0.50(0.76)	-0.1 ± 0.6	**0.005(0.07)**

[1] Statistical significance of within-group shifts (Δ week 6-baseline) were determined by Wilcoxon tests, while between-group shifts (Δ arabinoxylan vs. Δ microcrystalline cellulose) were determined by Mann–Whitney tests. *p* values were adjusted by FDR, whereas FDR significance was set at $q<0.15$. Δ SCFA, absolute change from baseline to week 6. BCFAs, branched short-chain fatty acids; SCFAs, short-chain fatty acids.
