# Supplementary material for: Gut microbiota modulation with long-chain corn bran arabinoxylan in adults with overweight and obesity is linked to an individualized temporal increase in fecal propionate
Source: Microbiome. 2020 Aug 19;8:118. doi: 10.1186/s40168-020-00887-w (PMC7439537; doi:10.1186/s40168-020-00887-w)
Supplement: Supplementary file 11 — Additional file 10: Table S4. Multiple linear regression analyses between arabinoxylan-induced fecal SCFA responses and bacterial features. Table provides the variables used for multiple linear regression analyses and corresponding results, which are presented as heatmaps in Fig. 7 and Fig. S5 [file 40168_2020_887_MOESM10_ESM.pdf]

Additional file 10: Table S4. Multiple Linear Regression Analyses Between Arabinoxylan-Induced Fecal SCFA Responses and Bacterial Features[1].

			**Fecal SCFA Concentration Response (Δ week 6 - baseline; dependent variables)**								
			**Propionate**			**Acetate**			**Butyrate**		
		**Predictors**	**β-Coef**	***p* value (adj.)**	**AICc**	**β-Coef**	***p* value (adj.)**	**AICc**	**β-Coef**	***p* value (adj.)**	**AICc**
**Baseline Microbiota**	All OTUs	PC1	2.86	**0.01(0.039)**							
		PC2			133.2	-4.15	0.03(0.056)	146.2	-1.21	0.05(0.07)	113.3
		PC3	2.77	0.03(0.06)							
	All CARGs	PC2	2.75	0.56(0.6)	141.1	-8.38	0.2 (0.24)	150	4.67	**0.02(0.048)**	111.3
	Sig. OTUs	PC1			134.5	-8.46	0.03(0.056)	150	-2.85	**0.02(0.049)**	115.6
		PC2	7.23	**0.02(0.049)**		2.30	0.58(0.64)		-0.80	0.52(0.55)	
	CARGs	CARG5	5.54	0.07(0.09)		10.16	**0.01(0.043)**				
		CARG6			139.0			144.2	-1.58	**0.014(0.043)**	110.3
		CARG7	1.43	0.03(0.056)							
	OTUs	OTU85 *B. obeum*	-21.86	0.03(0.056)							
		OTU46 *C. leptum*	-9.28	0.17(0.19)	139.5			147			110.7
		OTU41 *E. oxidoreducens*				-13.48	0.046(0.07)		-5.02	**0.016(0.047)**	
**Shifts of Microbiota (Δ week 1 - baseline)**	All OTUs	PC1	-2.66	0.03(0.06)							
		PC2	-2.48	0.07(0.09)	136.3			149.4			116.3
		PC3				3.35	0.14(0.17)		0.88	0.20(0.25)	
	All CARGs	PC1			132.13	-5.28	0.159(0.193)	152.9	-2.14	0.21(0.24)	120.62
		PC2	-9.57	**0.007(0.038)**		-7.22	0.33(0.375)		-0.038	0.98(0.98)	
	Sig. OTUs	PC1	-6.99	**0.011(0.039)**							
		PC2			133.2			144.4	2.67	0.08(0.10)	114.2
		PC3				12.00	**0.01(0.039)**				
	CARGs	CARG1	1.89	**0.0045(0.03)**	131.1			148.6			116.2
		CARG7				2.02	0.09(0.11)		0.482	0.23(0.25)	
	OTUs	OTU6 *P. copri*	-1.50	0.04(0.07)							
		OTU38 *P. succinatutens*	8.38	0.05(0.07)	138.67			140.2			103.1
		OTU7 *B. uniformis*				10.32	**0.002(0.018)**				
		OTU41 *E. oxidoreducens*							7.22	**0.0006(0.0097)**	
**Shifts of Microbiota (Δ week 6 - baseline)**	All OTUs	PC1	-4.23	**<0.0001(0.005)**							
		PC2	-1.86	0.046(0.07)	123.7	3.72	0.10(0.12)	148.7			118.0
		PC3							0.16	0.80(0.81)	
	All CARGs	PC2	-10.89	**0.001(0.014)**	128.95	-12.72	**0.018(0.048)**	144.95			108.98
		PC3							6.07	**0.007(0.038)**	
	Sig. OTUs	PC1	6.54	**0.0006(0.0097)**	125.0	8.05	**0.02(0.049)**	148.7			112.6
		PC2	6.40	**0.005(0.03)**		-3.92	0.35(0.38)		-3.14	0.039(0.067)	
	CARGs	CARG1	1.63	**0.0016(0.018)**		1.64	0.05(0.07)				
		CARG2			128.7	0.11	0.93(0.95)	151.6			109.4
		CARG3							2.13	**0.009(0.039)**	
	OTUs	OTU85 *B. obeum*	9.76	**0.007(0.038)**							
		OTU11 *Subdoligranulum* sp.			132.3	8.56	0.027(0.056)	145.8			108.4
		OTU41 *E. oxidoreducens*							8.48	**0.006(0.035)**	

[1] Each model contains the best one or two predictors of PCs, CARGs, or significant OTUs selected by stepwise regression. All models were adjusted by sex/fiber dose. Quality of each model was evaluated by corrected Akaike information criterion (AICc). adj., FDR adjusted *p* values; β-Coef: β-Coefficient; CARGs, co-abundance response groups; PC, principle component; Sig. OTUs: significantly responding operational taxonomic units.
